# Pregnancy intentions of young women in Canada in the era of climate change: a qualitative auto-photography study

**DOI:** 10.1186/s12889-023-15674-z

**Published:** 2023-04-25

**Authors:** Danielle M. Smith, Javier Sales, Aleyah Williams, Sarah Munro

**Affiliations:** 1 Department of Family Practice, Faculty of Medicine, University of British Columbia, Vancouver, Canada; 2 Centre for Health Evaluation and Outcome Sciences, Providence Health Care Research Institute, Vancouver, Canada; 3 Department of Obstetrics and Gynaecology, Faculty of Medicine, University of British Columbia, Vancouver, Canada

**Keywords:** Climate Change, Family Planning, Eco-Anxiety, Women’s Health, Sustainability.

## Abstract

**Background:**

Climate change poses a global health risk through consequences such as sea level rise, wildfires, and increased air pollution. Children born today and in the future may be disproportionately affected by climate change. As a result, many young adults are rethinking having children. The impacts of the climate crisis on the decision-making of parents is an understudied area of research. This study aims to be one of the first to explore how climate change impacts the pregnancy intentions of young women in Canada and their perspectives towards childbearing.

**Methods:**

We conducted auto-photography and qualitative interviews. Participants were recruited using social media, and were aged 18–25, nulliparous, assigned female at birth, and were either current or previous residents of British Columbia, Canada. We asked participants to take photos that responded to the question, “Show us how climate change impacts your decision to have a family,” then complete a virtual, one-on-one interview during which photo-elicitation was employed to guide conversation about participants’ decision-making related to childbearing and climate change. We subjected all transcribed interviews to qualitative thematic analysis.

**Results:**

We conducted in-depth interviews with seven participants who discussed a total of 33 photographs. Analysis of participants’ interviews and photographs identified themes of eco-anxiety, hesitancy towards having children, sense of loss, and a desire for systemic change. Participants experienced anxiety, grief, and loss when faced with thoughts of change associated with their environments. Climate change impacted all but two participants’ childbearing decision making, which was found to be interrelated with social-environmental factors, such as cost of living.

**Conclusion:**

We aimed to identify the ways in which climate change may impact youth decisions to have a family. Further research on this topic is needed to understand the prevalence of this phenomenon, and to build such considerations into climate action policy and family planning tools used among young people.

**Supplementary Information:**

The online version contains supplementary material available at 10.1186/s12889-023-15674-z.

## Background

Climate change is a critical health crisis. Its effects on the environment and communities are well-documented and include sea level rise, disruptions to food supply chains, and increased air pollution [1]. Recent research indicates that without aggressive action, children born today will be disproportionately impacted by climate change compared to older generations [1]. As a result of these potential consequences on future generations, along with slow and limited global policy action, young adults are putting pressure on governments through mass protests [2]. In addition, many young adults are rethinking decisions to have children in a future of uncertainty [3, 4]. Reports in The Guardian and BBC News illustrate how such movements as “No Future No Children”, “Conceivable Future”, and “Birthstrike” exist both in Canada and abroad and are composed primarily of young adults who claim that climate change is a large factor in their decision to have children [5, 6]. There have also been recent peer reviewed articles on this topic, highlighting increasing concern among individuals about having children in the context of climate change [7, 8] .

Much of the previous research investigating the intersection of climate change and childbearing primarily focused on family planning as a means to slow carbon emissions. For example, a previous study used analytical models and simulations to show that by choosing to have one less child, an American woman can reduce the sum of carbon emissions produced by her and her descendants by 9441 tons [9]. However, research regarding this “population factor” as a mechanism to curb climate change has been criticized for suggesting that women’s reproductive rights should be ignored to fight climate change [10]. Indeed, the idea of population control as a factor for mitigating climate change has drawn much criticism from global communities, including environmentalists [10].

There is scant empirical research available that investigates the effects of climate change on young women’s’ childbearing decision-making. Research on women’s decision-making to be childfree by choice has previously identified that the decision is typically highly contextual and due to several factors over the reproductive years rather than a single moment in time [11, 12], namely women’s concerns about finances and interruptions to education and career [13]. Some young women actively choose early not to have or raise children and stick with this decision throughout their life course, while others may be ambivalent or postpone childbearing until identifying as childfree by choice [14]. Studies on the role of climate change and reproductive decision making of young people topic have focused on two impacts: the first is to do with the desire to reduce ecological impact through forgoing raising a family, and the second is having concern over the quality of life of future offspring. A 2020 study conducted by Schneider-Mayerson and Leong highlighted how 59.8% of American survey respondents aged 27–40 reported being “very” or “extremely concerned” about the carbon footprint of childbearing [15]. In addition, many participants stated that their future children’s carbon footprint had led them to have (or plan to have) a smaller family [15]. The same study found that 96.5% of respondents were “very” or “extremely concerned” about the well-being of their existing or future children in a climate-affected environment. A Canadian study completed in Thunder Bay, Ontario also explored reproductive intentions and environmental thinking among university students, and found that environmental concern as measured by the “New Ecological Paradigm” was associated with a lower fertility intention [16]. Two recent articles involved interviews with adult couples in Norway and young adults in New Zealand and the USA regarding their intentions to be “environmentally childfree” in the context of climate change in order to eliminate the “carbon legacy” of reproduction [7, 8]. Both highlighted how climate anxiety and pessimism is increasingly affecting peoples’ decisions to bear children [7, 8]. Interestingly, there is conflicting data about the effect of gender on reproductive decision making in the era of climate change. In the survey conducted by Schnieder-Mayerson and Leong, there was no statistically significant difference between the eco-reproductive concerns of men and women [15], whereas a recent Swedish survey study with more than 1300 participants found that women, regardless of parenthood status, were more concerned than men about climate change and its effects on future generations [17].

There are many other reasons why climate change may impact the decision to have a family. The climate crisis has been linked to repercussions on mental health such as depression, anxiety, and following severe climate change-related events, post-traumatic stress disorder (PTSD) [18–20]. The term “eco-anxiety” has been used to describe a debilitating worry about current and future losses related to climate change [19] and one large international survey study found that climate change and government inaction are chronic mental health stressors in the lives of young people that may have considerable, permanent, and negative impact [21]. Further research has identified certain populations in North America that are already facing mental health repercussions of climate change. For example, a multi-year, community-driven case study situated in the Inuit community of Rigolet, Nunatsiavut, Canada, illustrated how the Inuit are disproportionately affected by climate change and feelings of loss associated to changes to their environments [22]. Other qualitative research indicates there are increased reports of PTSD in individuals that experience extreme weather events, such as hurricanes and wildfires [23, 24]. Despite this emergent body of literature, there is limited research on youth in North America regarding their childbearing intentions in the context of climate change.

The decision to have a child is multifactorial, and the implications of the climate crisis on childbearing decision is understudied, especially in Canada. We aimed to answer our guiding research question: What are the thoughts, feelings, and perspectives of young women who are capable of becoming pregnant (including transgender men, non-binary folks, agender individuals, and cis-gender women, among others) in British Columbia, Canada, toward having children in the context of the climate crisis? Our study builds on previous qualitative research on this topic by adding an auto-photography approach to discuss perspectives related to reproductive decision making during the era of climate change, in a cohort of young women who have never become pregnant.

## Methods

In this study we used auto-photography to explore participants’ perspectives towards future pregnancy and parenthood in the context of climate change. Auto-photography is an ethnographic method that employs visual methodologies to understand the perspectives of research participants with respect to a specific research question [25]. This visual methodology is unique as it provides researchers a view into how participants perceive their environments and allows participants to speak for themselves. This method has three relevant advantages for answering our research questions: photos act as tangible stimuli that help us and participants understand their unconscious understanding and use of representations, images and metaphors; it leads to different and richer information than other methods; and it can help to reduce the power differential between researchers and participants, by promoting an interviewee-led style of interview and centering their experiences and knowledge [26].

This study took place in British Columbia in the summer and fall of 2020. In accordance with local public health recommendations in the context of the coronavirus pandemic, this study was conducted using virtual software. As such, there were no restrictions on participant location within British Columbia.

### Sample

Participants self-recruited to the study using social media platforms (Twitter, Facebook, Reddit) on which the study was advertised. The posts invited participants to contact the research team for further information on the study. Participants were eligible if they were between the ages of 18 to 25, nulliparous, assigned female at birth, had resided in British Columbia at any point in the past five years, and spoke English. We did not collect participant demographic characteristics for reporting purposes in this study to protect the anonymity of our sample. The decision to sample individuals in British Columbia was deliberate; we wanted to explore the experiences of individuals within a similar socio-political and geographic region in Canada. Climate experiences differ substantially between British Columbia and other geographic areas in Canada. For instance, British Columbia has been impacted by a unique and interrelated series of seasonal wildfires, flooding, and heat waves for several years. In contrast, its neighbouring province, Alberta, is in a different physiographic region and has not experienced these specific climate events to the same degree. Consistent with a qualitative approach to seek a relatively small sample of people who have common climate change experiences, we limited recruitment to individuals in British Columbia, or who have lived in British Columbia in the last 5 years. We sought to include the perspectives of people who were born with the ability to become pregnant, including trans men, non-binary folks, agender individuals, and cis-gender women. One of our aims was to generate knowledge that could inform approaches to family planning for people capable of becoming pregnant.

We used purposeful sampling to collect, analyze and share diverse attitudes, beliefs and perspectives among young women towards childbearing in the era of climate change. Braun and Clarke’s (2022) approach to reflexive thematic analysis recommends avoiding claims of ‘saturation’ [27]. We followed their guidance to instead consider the ‘information power’ of our dataset. Conceptualized by Malterud, Siersma and Guassora (2016) this involves reflections on the information richness of the dataset and how that meshes with the aims and requirements of the study [28]. The more information a qualitative dataset holds, and its relevance for the study, the fewer participants required to meet the study aims. We assessed the information power of our sample continuously through discussion and consideration of the core domains of information power (aim, specificity, theory, dialogue, analysis).

### Data collection

Participants that responded to the social media posts were provided with information on the study and provided consent forms for participation. Eligible participants were asked to take up to five photographs and rank them in order of least to most impactful in response to the statement: ***“Please show us how climate change impacts your decision to have children, then rank these photographs from least to most impactful.”*** Our consenting process explained that taking five photographs and reflecting on their impact may take participants up to 90 minutes to complete; in total, participants were expected to dedicate two and a half hours toward participation (photo-taking and interview time combined) for which they were compensated at a rate of $20 per hour with a maximum total honorarium of $50.00. We selected a limit on photos to ensure sufficient depth in the interview (rich discussion of the photos), rather than breadth. Participants were asked to anonymize facial features of individuals, including themselves, if present in their photos. No photos were excluded from the study. Once the study team received the photos, participants were scheduled for interviews using virtual video-conferencing software (Zoom). Participants then completed a 45-minute photo-elicitation interview using open-ended interview questions adapted from Lin, *et. al.* (2017) (Supplementary file 2). Study team members DS and JS, who had no prior relationships with any of the study participants, conducted interviews. Interviewers used photo elicitation techniques [29–31] to analyze photographs with participants to create rich, collaborative interview data. Participants guided the interviewer through their interpretation of their own photographs, minimizing the need or ability for the research team to erroneously interpret the photos. Autophotography was chosen specifically to empower the participants with the ability to express themselves through art and use it as a subjective tool to facilitate deeper disclosure. With each participant, we sought to explore different layers of meaning through this method, from latent, visual descriptions of each photo to interpreting what each represented, including participant emotions, memories, and ideas. Interviews were audio-recorded and transcribed using a Canadian transcription service (Advanis).

### Analysis

We de-identified the transcripts and assigned a numeric identifier for each participant. We identified and developed themes related to the study question following principles of reflexive thematic analysis [32]. We did not further analyze participants photos on our own. Study authors SM and AW independently coded the same sample of transcripts (n = 3) to create preliminary codebooks. Our coding process was four-fold: first, we conducted open and in vivo coding to identify properties of emerging concepts, second, we used focused coding to identify and organize codes into smaller batches of related phenomena, third, we compared data to data within codes using constant comparison, and fourth, we used theoretical coding to sort, synthesize and organize the data into major conceptual categories, which we then discussed and collated into a coding framework [32]AW utilized this framework to code all interviews in NVivo for Windows, and shared her results with DS and JS who formatted them into the manuscript. Throughout the research process we engaged in verification strategies to promote reflexivity, including keeping an audit trail, and practicing self-awareness of our identities compared to those of our participants. Photo elicitation interviews contributed to the trustworthiness of our findings as a member checking technique. We also met through multiple team meetings to critically reflect on the data and discuss our assumptions and possible biases.

## Results

Seven cis-gendered, English-speaking women (age range 18 to 25) participated in interviewees and shared a total of 33 photographs (Supplementary file 2). Our analysis of photo-elicitation interviews identified six key themes related to climate change and childbearing: planning for a “dire future,” experiencing anxiety, feelings of loss, catalyzing events, feeling like an outlier, and calling for systemic change.

### Planning for a “dire future”

**Fig. 1:**
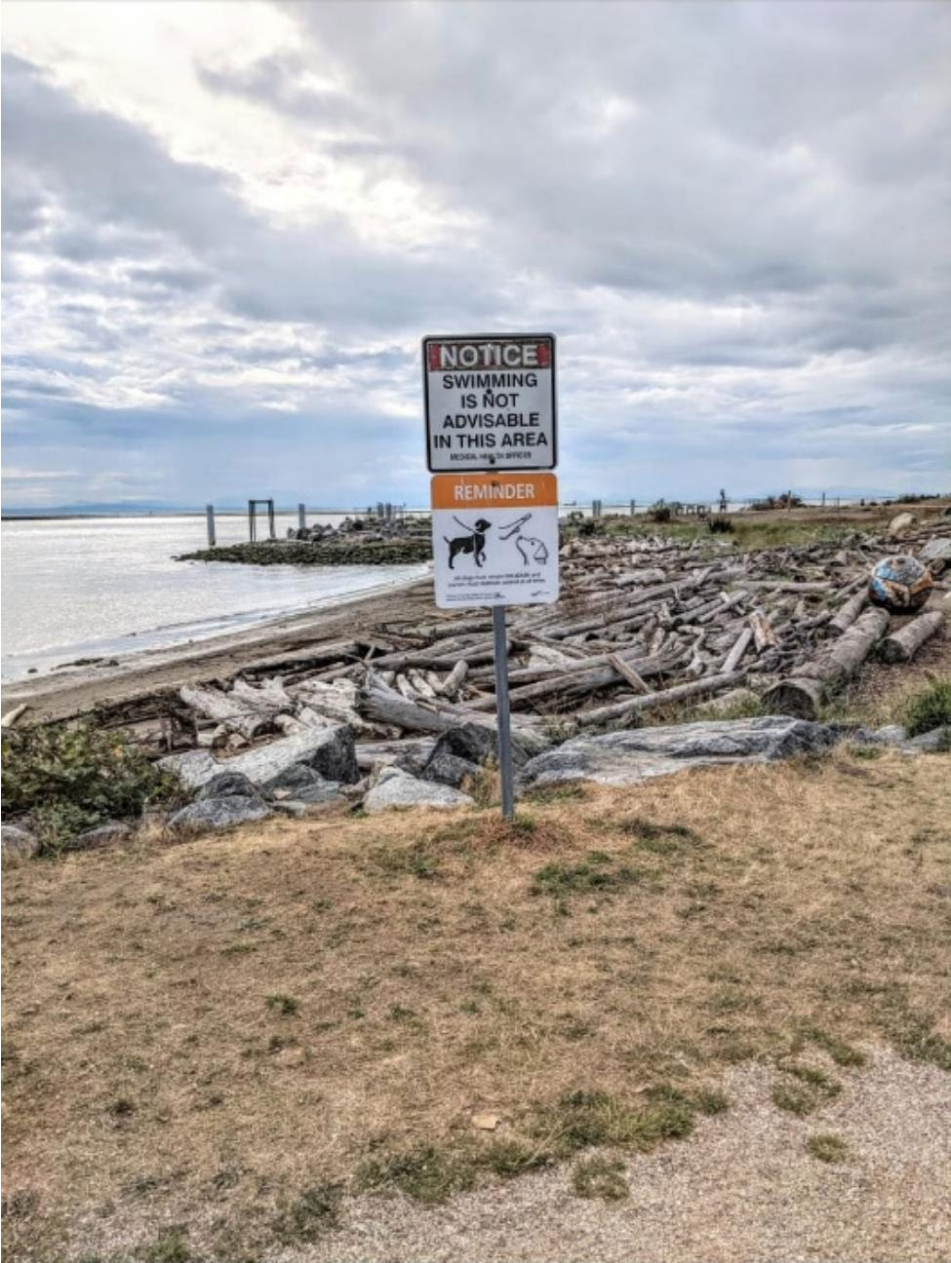
*Contaminated water*, Participant 2

Six of the seven participants stated that climate change either has already affected or may affect their decision to have children. Two individuals stated they would not have children due to climate change alone, with one sharing, “if I suddenly get a bunch of news that new climate change policies have been enacted all over the world, and there’s proof that it’s being enforced, then I’ll be hopeful and perhaps change my stance on having children in the future, but for now I wouldn’t want to have children because of the dire future that I’m predicting.” Similarly, another participant said, “I don’t think I want to have kids because I could not provide that experience with nature in the outdoors because of the state of the world…and I’m very much a kid and babies person” (see Fig. 1). Additionally, two participants reflected on inequity as a contributing reason why they or their peers were uncertain about having children. One woman said that her decision to become a mother in the next five to seven years, what she referred to as “continuing a blood line”, was influenced by the inequitable consumption of resources in Canada as compared to developing nations. The second woman reflected that “even people who would like to have children answer that question with trepidation because of growing inequality” as well as “environmental degradation and the uncertainty of the kind of world [their potential children] would be living in.”

In contrast, two participants decidedly wanted to have children despite the effects of climate change. While acknowledging the importance of climate change, one participant shared, “[My partner and I] had this discussion, and there’s a lot of valid points either way but climate change is not one of the reasons why I wouldn’t have a child.” Her partner studied sustainability and told her having children was “not the best idea”. The other participant who planned to have a family in the future said that “the climate condition doesn’t really play a key role for my decision” however “if it became more severe then I may change my decision.”

While most participants grappled with climate change as an important factor in their reproductive decision making, they also spoke to other factors. These included their age and perceived life stage, the presence of a supportive partner, emotional support, and financial stability: “It’s important to have a really strong bank account because you are caring for a dependent, and you have to get food on the table.”

### Experiencing anxiety

**Fig. 2:**
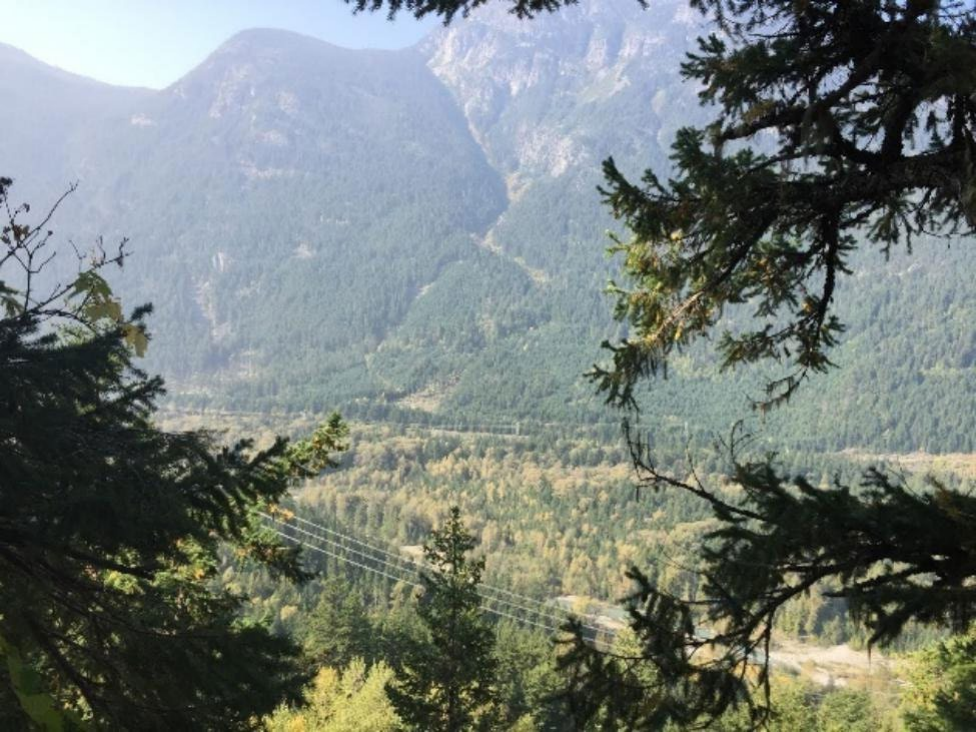
*Hidden beauty*, Participant 7

Almost all participants characterized their feelings as “anxiety” and dread when discussing a future affected by climate change. Participants expressed negative feelings about current events related to climate change, using multiple negative adjectives such as “unsettled,” “gloomy,” “concerned,” “anxious,” “afraid,” and “helpless.” As seen in Fig. 2, participants connected these emotions with photographs they took of the wildfire smoke that affected large swaths of the Pacific Northwest, including Vancouver, during the summer of 2020 (see Fig. 2): “I felt scared. One, we couldn’t go outside already because of pandemic, but now we can’t even get any fresh air because of the smoke, so I felt very restricted and a bit helpless.”

Some participants alluded to feeling powerless against the increasing rates of extreme weather events. When reflecting on their potential futures, some expressed feelings of a lack of control: “It feels very much out of my hands. There’s definitely times when I feel kind of helpless. Like you can do what you can do in your sphere of influence. And beyond that, it feels like I’m just shoved down into the void.”

### Anticipated feelings of loss

**Fig. 3:**
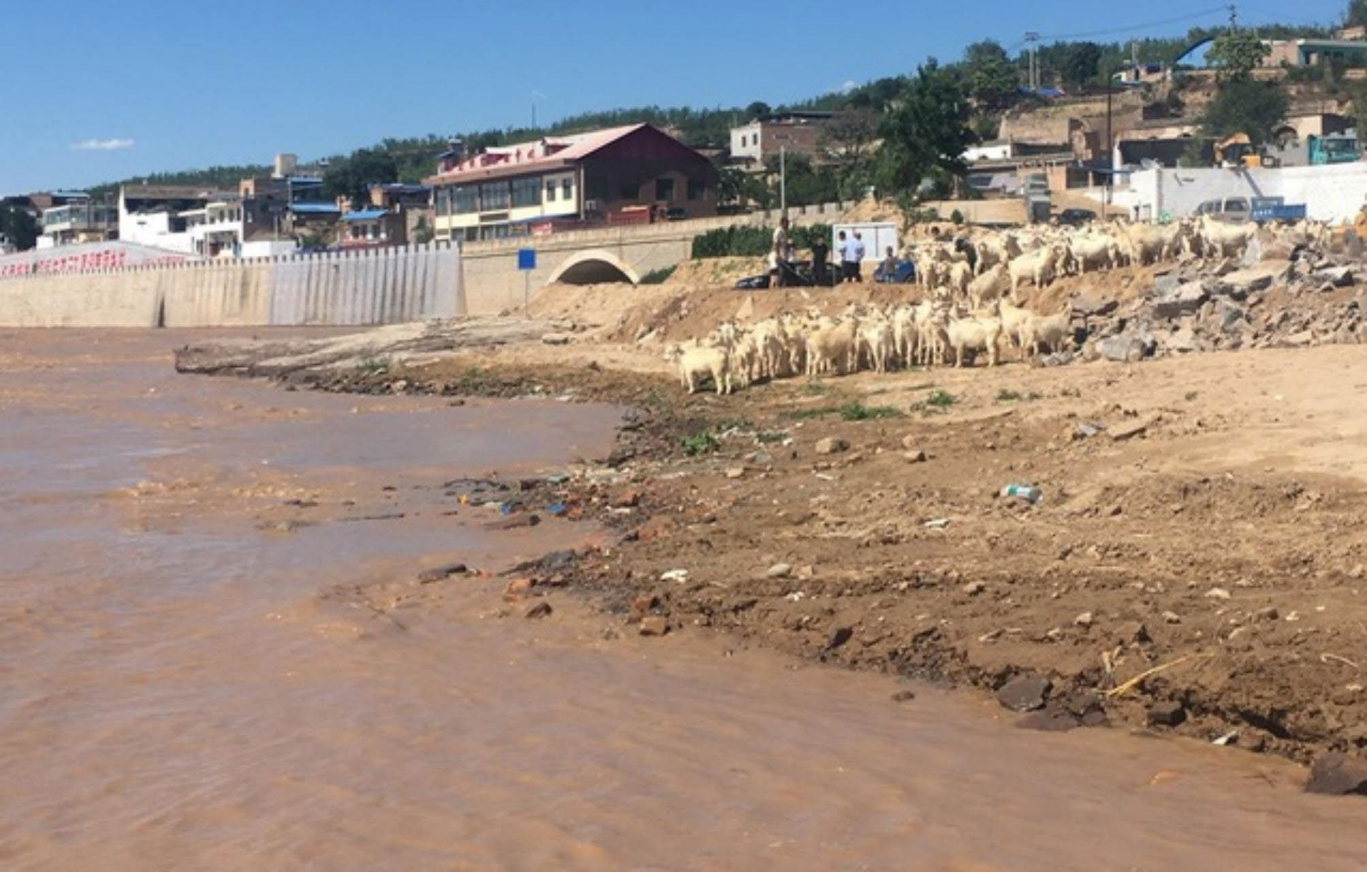
*A place it used to be*, Participant 4

Many participants spoke about preserving nature and wanting to share it with their future children. They expressed a sense of loss or disappointment when reflecting on aspects of the environment that they may no longer be able to share with current and future generations of youth: “Having nature accessible, like I’ve had it accessible in so many different ways, kayaking or hiking or snowboarding. I don’t foresee […] future generations having that nature accessible to them the way I have had it.” The feeling of sadness was pervasive across multiple interviews and often featured places that figured prominently in the participants childhoods. As one participant reflected, referring to Fig. 3, “My dad would tell me stories of swimming across the river or playing in the river. It seemed to at least figure prominently in his childhood. And it makes me really sad to hear that this river is shrinking or receding and becoming more polluted.”

### Catalyzing events

**Fig. 4:**
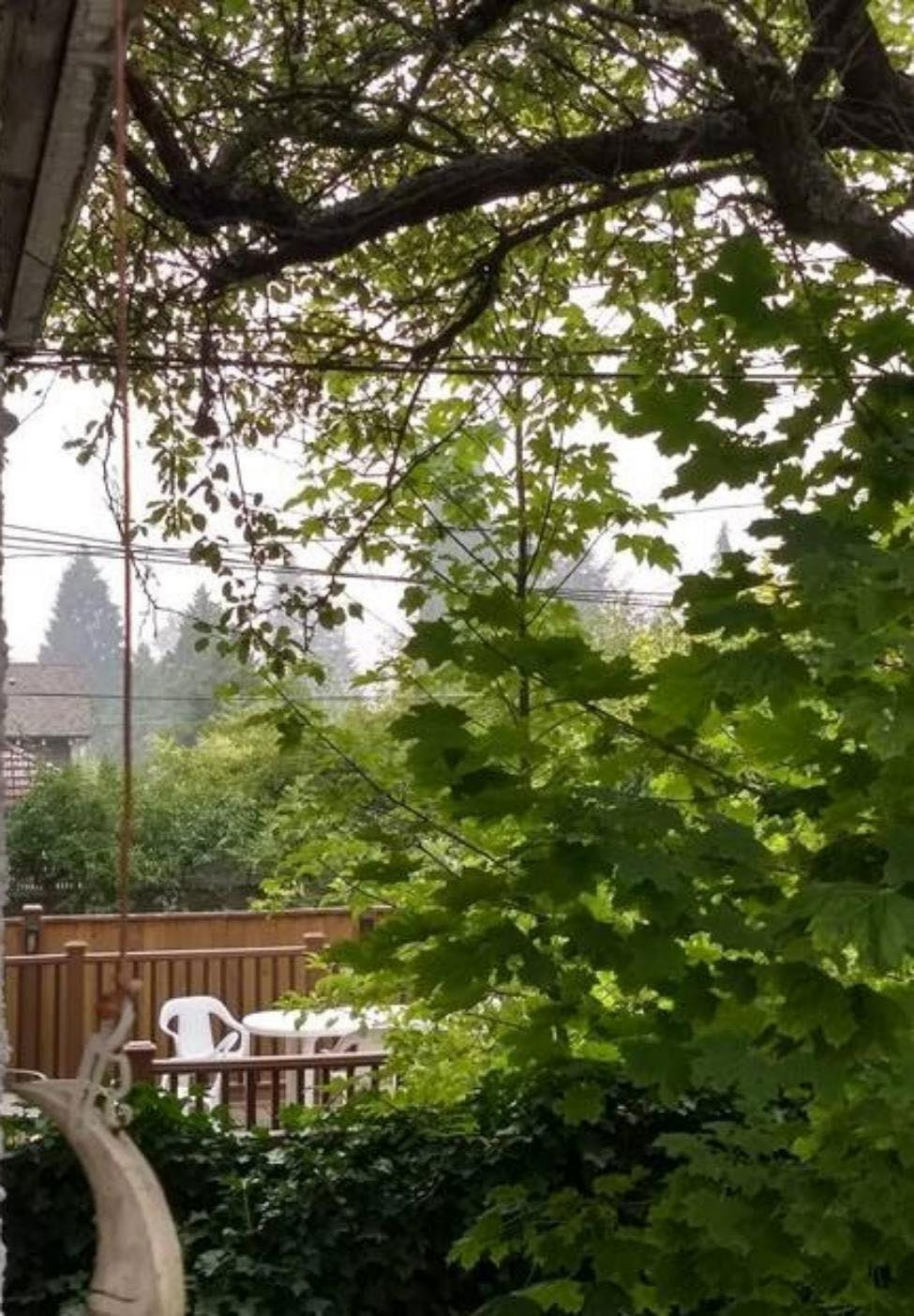
*Wildfire smoke*, Participant 3

Some of the participants identified specific life events or experiences that influenced and led to a change in their perception of climate change and childbearing. To one participant, the effects of last year’s wildfires was a new experience that provoked reflection through Fig. 4: “I’ve never experienced this extent of an environmental crisis directly within my sphere of the world, so it was really shocking to see this actually impact my personal life and made me reflect how my life will be in the future.”

**Fig. 5:**
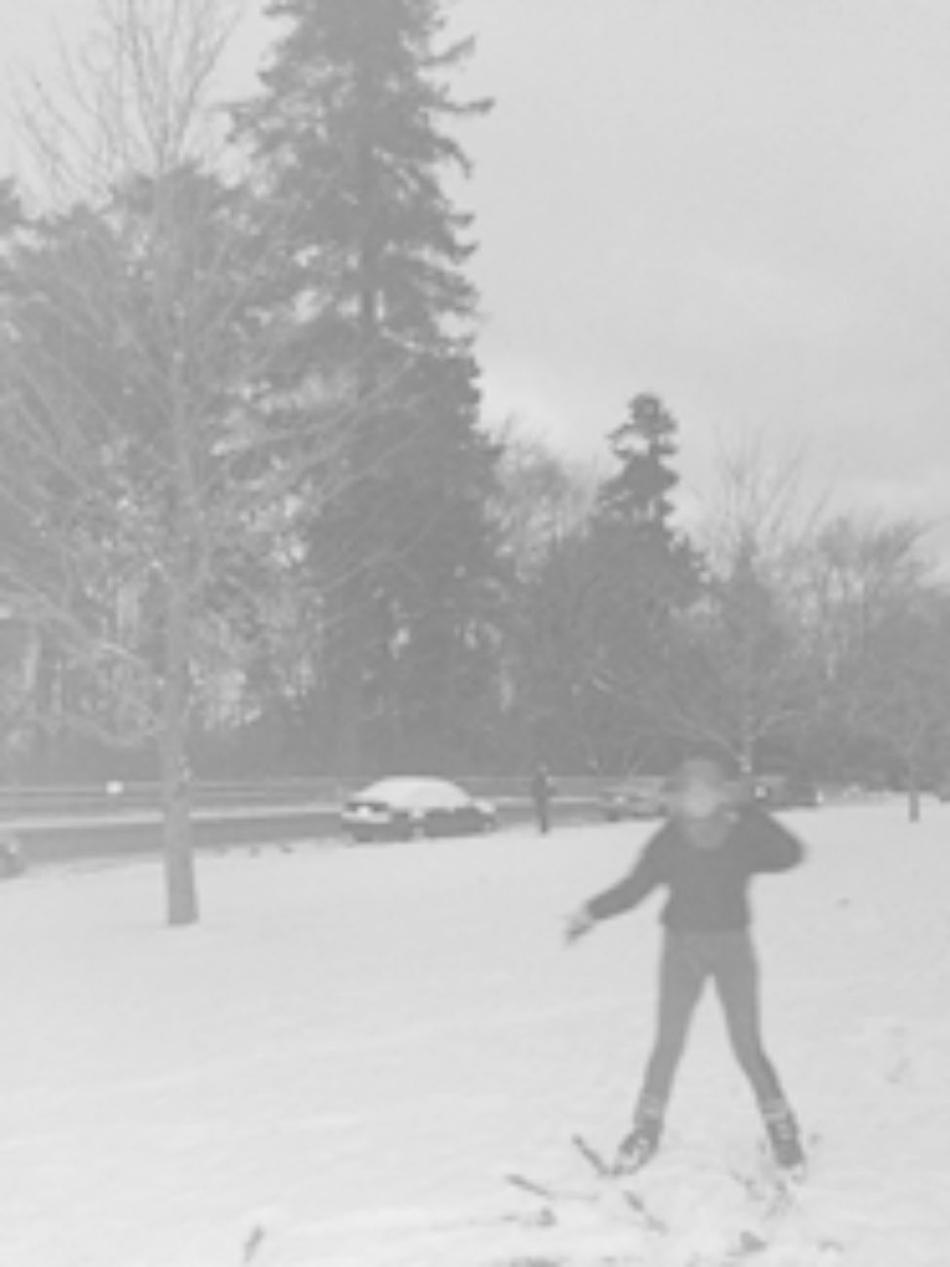
*Trying new experiences wherever you can*, Participant 5

Other important events included schooling, experiences with family and friends, and exposure to environmental advocacy such as climate strikes and nature. One participant spoke of the impact of being in a community which introduced her to nature-based activities, and reflected in Fig. 5 on the differences between that and her life before moving to Canada from the United Kingdom: “This photo reminds me that I came from a very small place to a very big place, and when my eyes were opened to more extreme conditions and other ways of living.”

Participants spoke to the significance of these events with respect to their understanding of climate change. Indeed, one participant noted that exposure to university made her feel less “ignorant” and opened her eyes to the impacts of climate change.

**Fig. 6:**
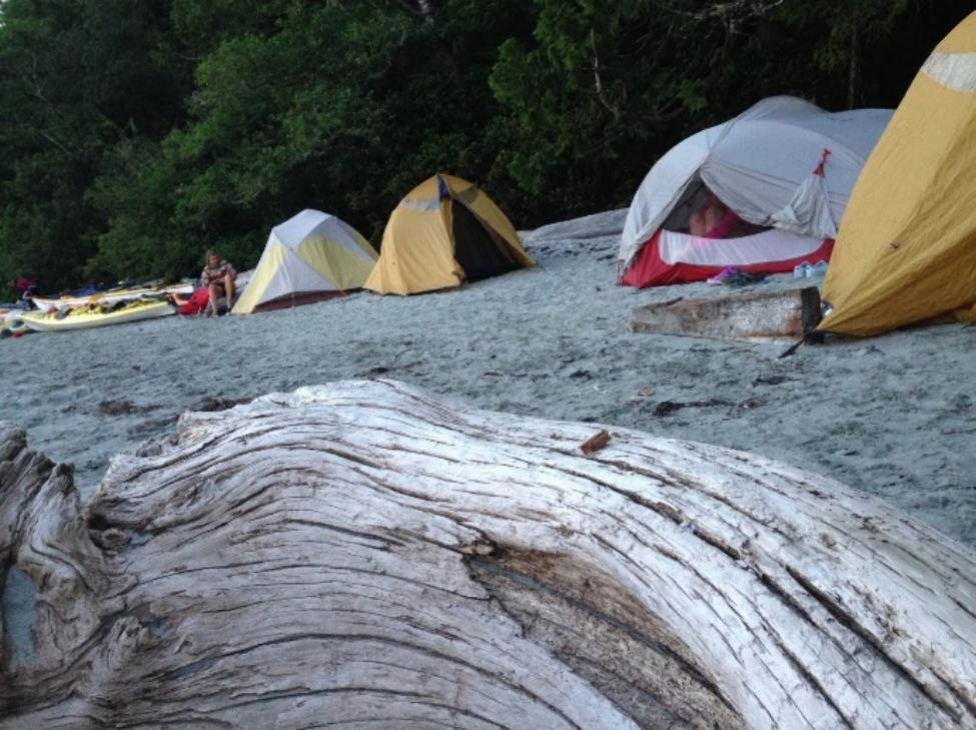
*Untitled*, Participant 7

Another participant was able to reflect on being in nature and her “epiphany” while on a school organized trip (Fig. 6). To this participant, this experience reinforced not only their desire for future offspring to have the same experience, but also their stance toward protecting nature.

**Fig. 7:**
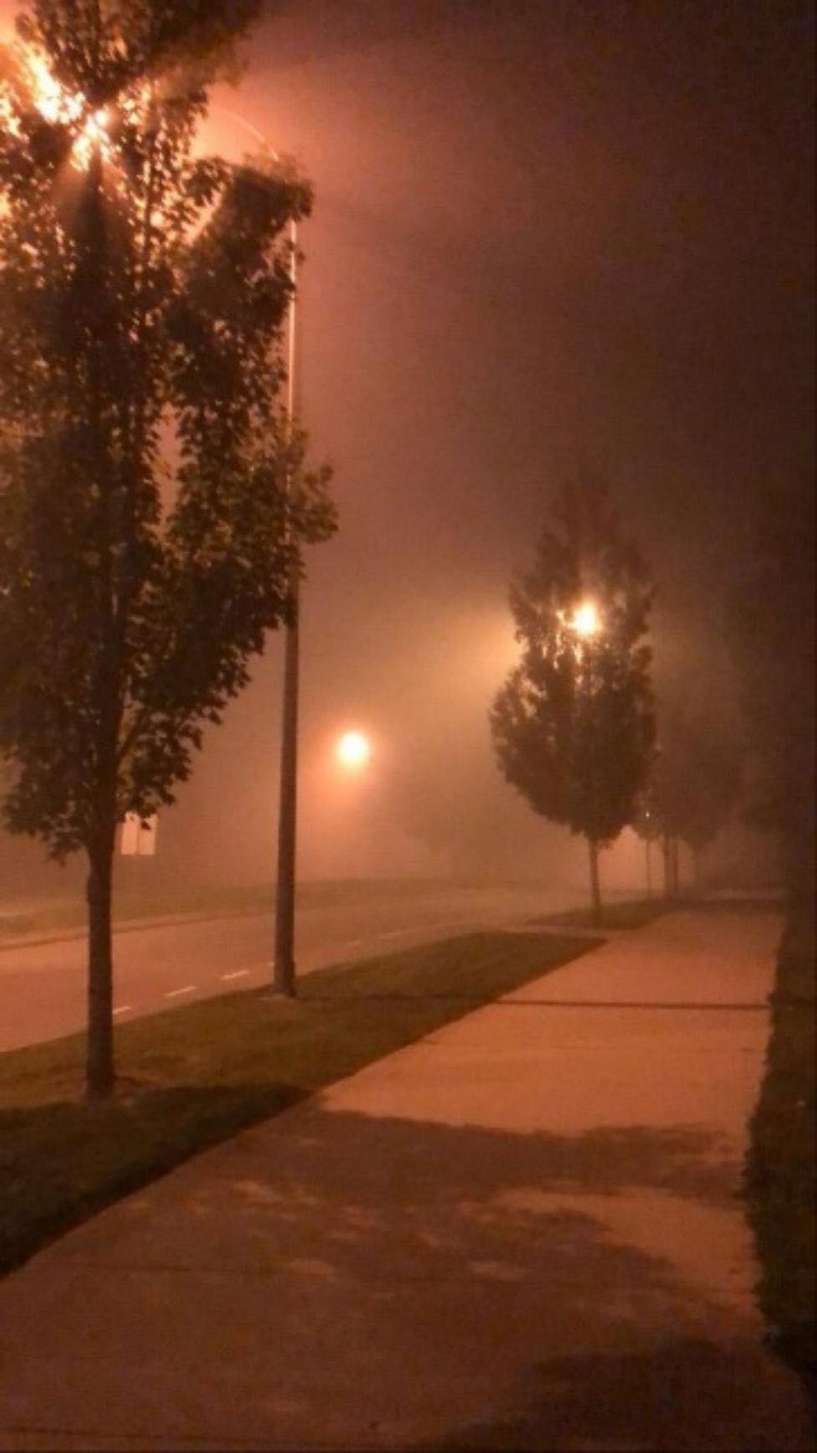
*How far can you really see what’s ahead of you?*, Participant 5

Coincidentally, this study was conducted over a period where the California, Oregon, and Washington wildfire smoke spread into Vancouver and surrounding areas may have impacted feelings of anxiety for many participants. Indeed, four out of seven participants shared photos highlighting smoke or fog, and how they would not want to share a future impacted by these events with their offspring (Fig. 7).

**Fig. 8:**
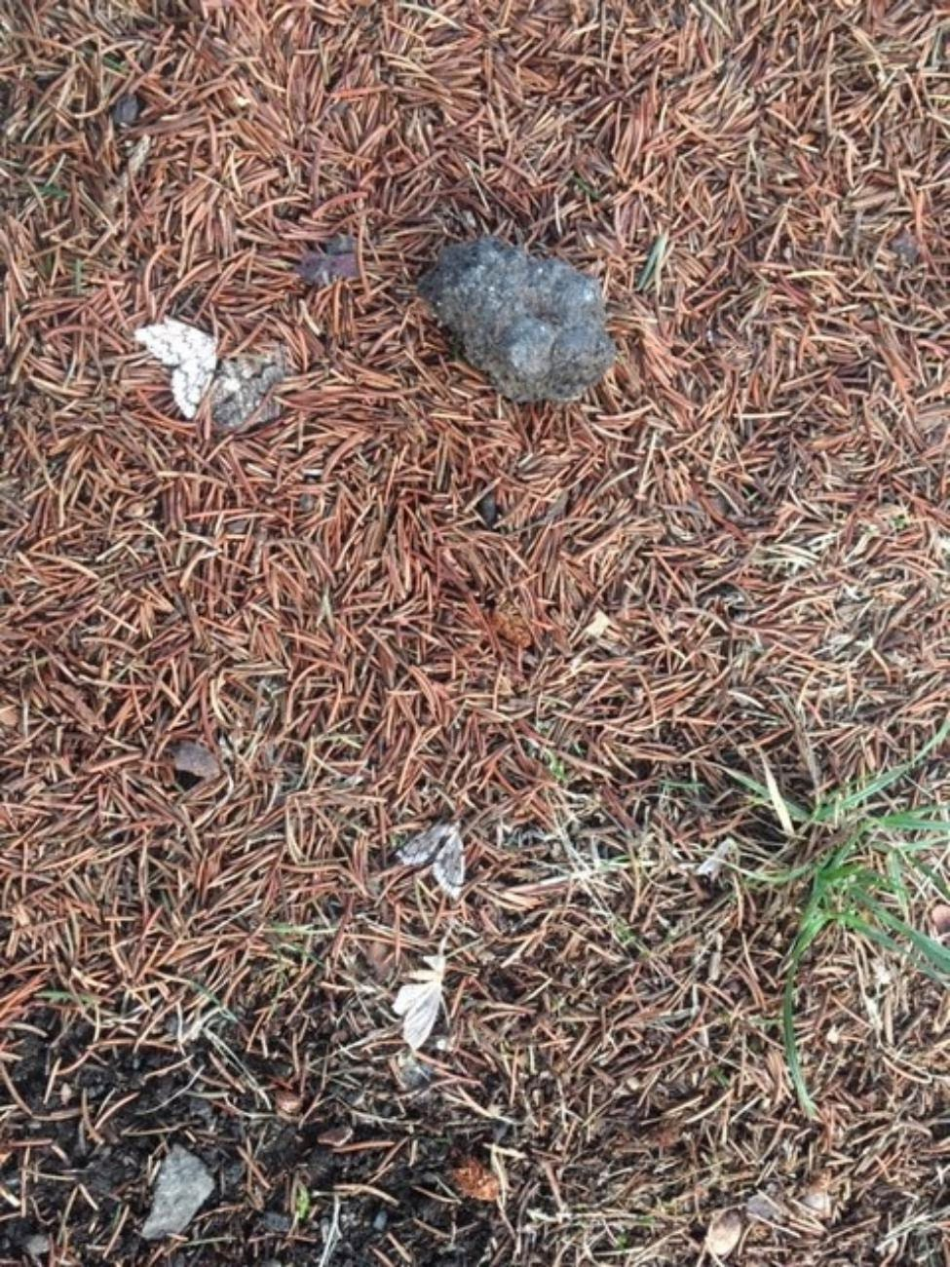
*Lord of the Moths*, Participant 4

While the wildfire smoke was featured prominently, participants shared photos that highlighted other weather and weather-related events that made them feel unsettled, including heavy snow falls and moth outbreaks. One participant reflected on their photo of the Looper moth outbreak that was brought on by prolonged hot weather in Vancouver in summer 2020 (Fig. 8), “It seemed like a pretty out of whack or unnatural or really freak phenomenon.”

### Feeling like an outlier

Participants shared feelings of a divide between themselves and other members of their families, older generations, or even other peers with respect to childbearing. As one described, “It was a group of four and all of the other three girls were really set on having kids, so when I shared that [I wouldn’t want to have kids], they were just shocked.” Another young woman spoke to the societal pressures places on women to have children, and how that conflicted with her desire not to child bear, “I don’t have a strong desire to produce offspring but feel like it would make my family happy and it’s a social expectation of women.”

Others felt supported when close associates or other family members felt similarly towards having children in the context of climate change: *“*My sister said, ‘Oh, I’m scared for what the future will look like and what the people in the future are going to be like and what they’ll have to suffer through […]’ I [said] probably wouldn’t have children because of that, and they were actually pretty content with that, like, understanding of it, which I guess is a little surprising.”

**Fig. 9:**
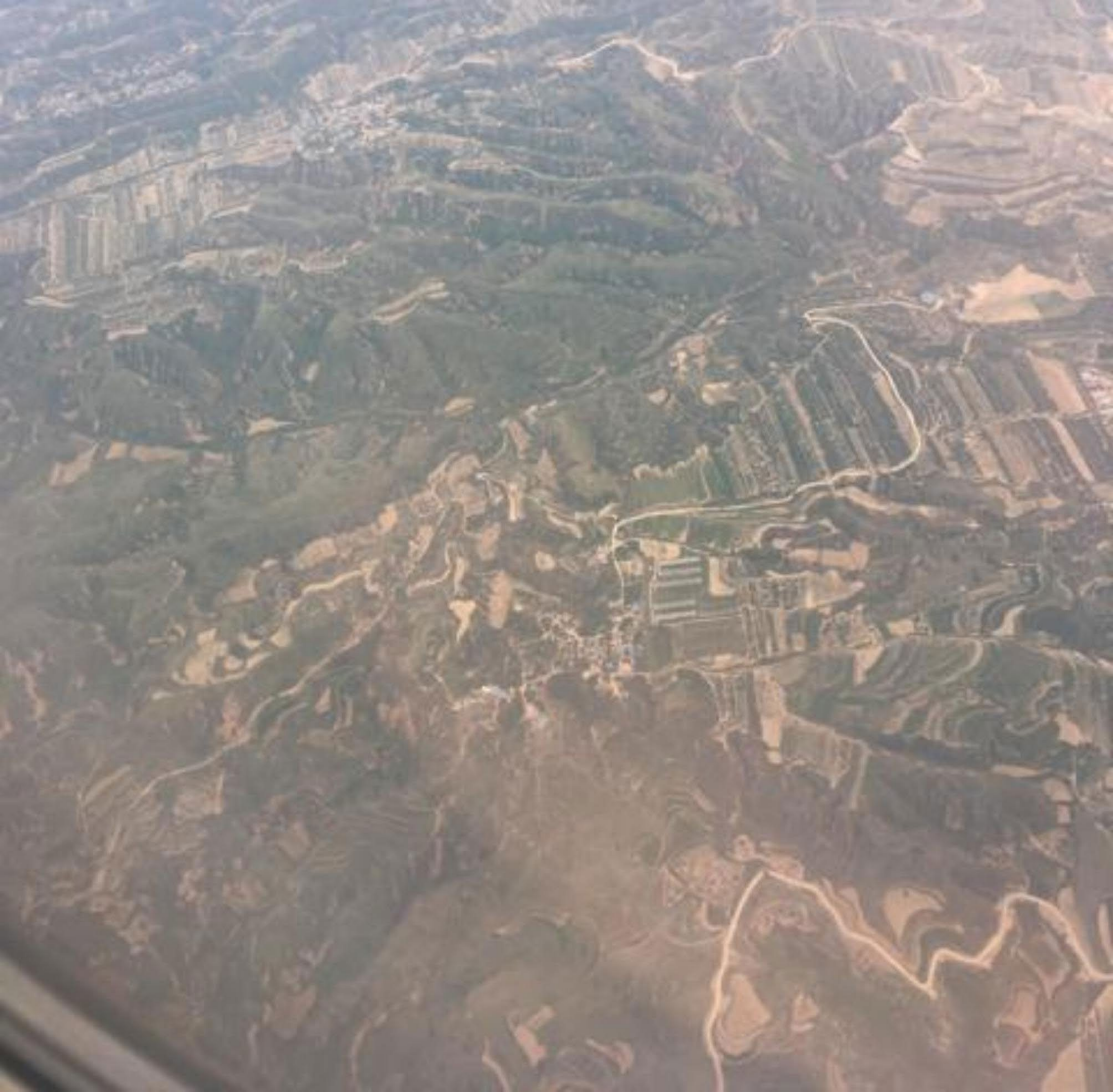
*The land bears scars*, Participant 4

When reflecting on landscape that had been changed by logging and developmental projects (Fig. 9), one participant referred to a divide between younger and older generations with respect to climate change: “I think there is a big gap or disconnection between the younger generation because, compared to the older one, because I feel like my parents or my parents’ generation didn’t really take climate change that seriously.”

### Calling for systemic change

Two of seven participants did not want to have children unless systemic change to address climate change occurred. Participants shared potential plans to tackle the climate crisis at an individual level and the adaptive behaviors they would want to share with their future children. These included passing down behaviors that are beneficial to the environment such as recycling, eating less meat, and, limiting their family to two children: “So even, whether that’s recycling or buying vegetables that aren’t in plastic packaging and things like that, but also not buying loads and loads of fast fashion. I don’t do that anymore. I choose sustainable brands.”

**Fig. 10:**
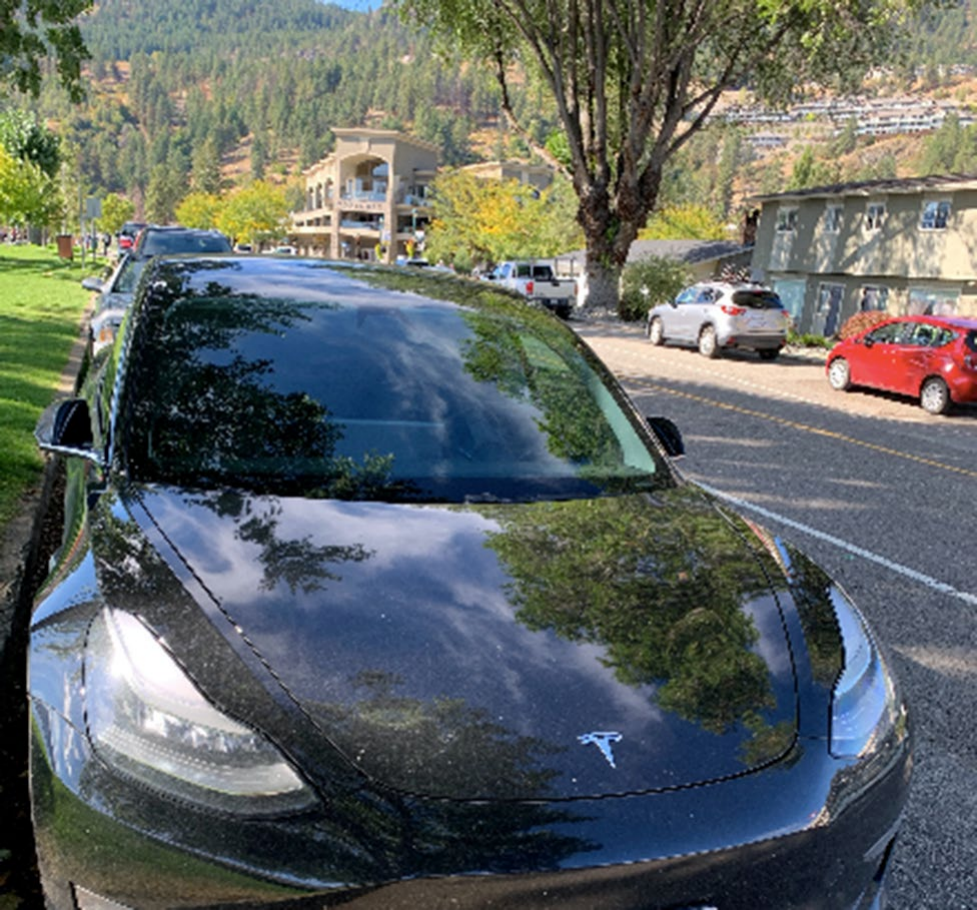
*Material innovation*, Participant 1

Many spoke directly to the need for systemic, global coordination and change that, if enacted, would change their decisions to have children in the future: “I think if there were governments in place that prioritize climate change and the environment, and it seemed like that was a priority globally. I think that would make me change my mind.” One participant in particular spoke to a feeling of hope and encouragement when thinking of new technologies and innovations that are being created to tackle climate change (Fig. 10). They described feelings of their small actions in comparison to corporations, like the Tesla electric vehicle manufacturer, which may contribute a larger impact on climate change.

## Discussion

This study highlights the variables that young women may consider when deciding to have children in the era of climate change. Participants’ interviews and photographs illustrated eco-anxiety, feelings of loss, hesitancy towards having children, and a desire for systemic change. However, while climate change was a consideration for many participants in their childbearing decision making, it was not the sole determining factor.

Young women in our study spoke to a hesitancy towards having children because of climate change, and often reflected on environmental changes that were already occurring in the present day. Many of the photographs shared, such as Fig. 2 “A place it used to be” were of locations tied closely with participants’ childhood and highlighted strong place-based ties to their identities and values. Participants discussed these photographs with a sense of loss and sadness because their future children may be unable to enjoy these experiences because of climate change. This notion of loss associated with changing environments has been found in previous studies, such as the work by Cunsolo et al. exploring the negative effects of climate change on members of an Inuit community in Labrador [22]. Their study highlighted the connection between a person’s place and overall well-being [22]. Indeed, the term “solastalgia,” previously defined in literature as “the pain or sickness caused by the ongoing loss of solace and the sense of desolation connected to the present state of one’s home and territory”[33] was highlighted by participants in our study as they reviewed their photographs. Future studies on this topic may benefit from identifying other ways in which changing landscapes influence the complex decision making related to childbearing among young women and youth in general.

Our findings add to recent qualitative research on the impacts of climate change on childbearing in young adults [7, 8]. A recent study by Nakkerud [7] highlighted how singles and couples in Norway experienced childbirth decision-making as a choice “in development” – making the choice, sharing (disclosing) the choice, and integrating the choice into one’s life and relationships. However, that study focused on a population of adults who were actively making or had made childbearing decisions, and the authors used a semantic analysis approach to describe their choices. Our study sought to go beyond description to provide interpretive insight into the motivations for childbearing decisions among a younger population of women who had not yet had children, and how those choices intersect with other considerations, like cost of living. Research by Helm et al. [8] involved an approach similar to our study and involved semi-structured interviews with 24 adults aged 18–35 in the USA and New Zealand to explore the role of climate change considerations in the formation of reproductive attitudes and motivations for going childfree. That study reported participants’ motivations for childbirth decision-making were related to societal level climate challenges, namely overconsumption and overpopulation. In contrast, data from our study highlighted the relationship between participant motivations and their personal, embodied relationships with the environment – how climate change will impact a child’s ability to swim in a cherished lake or go camping with other students. These experiences were characterized by a feeling of grief, as participants anticipated that the experiences they had as children would not be possible for their own children because of climate change. Our use of autophotography may explain why our data focus on personal, rather than societal motivations for childbearing preferences, because the arts-based method involves the generation and interpretation of images that best represent the individual participant.

Furthermore, our research contrasts other research on this topic by Bodin et al. which highlighted that while climate change was an important factor for many, it ultimately did not have a major impact on childbearing decision making [34]. Their study conducted focus groups with participants ranging from 17 to 80 years old, across genders, and parenthood status [34]. They reported that many participants found ways to combat their climate-related worries by either making more eco-conscious choices in other facets of life or having less children [34]. Our participants were younger than 25 years old and therefore more removed from the decision to have children. Indeed, the average age of childbearing in BC is the highest in Canada, at an average of 31.6 years old [35]. While Bodin et al. did interview a wide age range in their study, there was no distinction between the anxieties, concerns, and decision-making of the younger adult groups compared to the similarly nulliparous older adults [34]. Interestingly, some young women in their study noted the pressure placed on them by society to have children [34], which was similarly reflected by two participants in our study as highlighted in the “feeling like an outlier” theme. Having children is still considered one of the most defining aspects of femininity and womanhood, and the pressures placed on women to become mothers is well documented [36]. It would be interesting to compare climate change related decision making between nulliparous younger adults and those who are older, as older adults may negotiate increasing pressure to decide whether to start a family from their families and partners, which may reduce the importance of climate change on their childbearing decision making.

By combining autophotography and photo elicitation techniques, our study explored how key experiences and emotions can influence individual attitudes toward childbearing and climate change. The photos shared by our participants often evoked reflection on their experiences in nature, which stirred feelings of anxiety and fear for a future where this sort of connection may not be possible for their children. Indeed, eco-anxiety was a prominent theme in this study and was pervasive in almost every interview. This notion that youth are being faced with existential realizations with respect to climate change is something that is shared among other peer-reviewed literature on this topic [7, 8, 18]. The effect of climate change on youth mental health has been highlighted in other studies which show that climate associated fear and distress may negatively affect overall mental health among youth [37, 38]. This further highlights the need for action on climate change as it continues to affect youth wellbeing, and ongoing research on this topic is needed to quantify the effects of climate change on mental health.

While feelings of loss, grief, and anxiety featured prominently in our results, there was hope and optimism too. Reports in climate change literature highlight the importance of hope as a vital requirement for youth to feel motivated for action and change [18, 19]. The recent increase in global marches, school strikes, and advocacy movements led by youth highlights the increasing desire for systemic change and advocacy among today’s youth. The degree to which climate change policy action affects youth desires to have children is not currently well understood. Whether participation in these global advocacy movements affects young people’s decisions to have children would be interesting to explore in future research.

Our results may have implications for contraception counseling and family planning, with regard to climate change and associated worry. For young women seeking contraception to avoid pregnancy in the context of climate change, health care professionals should elicit their patient’s values and provide tailored information on contraceptive options that match the patient’s informed preferences. Shared decision making is a patient-centred model of information exchange that can support contraception providers [39], for instance in addressing difficult questions related to climate anxiety and in responding to ambivalence about family planning preferences. This model of information exchange aims also to assist the patient to clarify their specific preferences at that time and whether they are biomedical (prevent pregnancy), social (reduce ecological impact), emotional (manage anxiety about unplanned pregnancy), or related to overlapping concerns about cost and educational attainment [39]. This clarification provides the health care professional with an understanding of the patient’s information needs, which can support in identifying a course of action that matches the patient’s values.

We acknowledge some strengths and limitations of this study. First, our participants were a geographically restricted sample of seven cis-gender women. Our results will be applicable and adaptable to women in similar geographic and sociodemographic environments. Our purposeful sample allowed for a rich description of participants’ lived experiences, emotions, and perceptions of childbearing and climate change. To that end, our study may allow for a more detailed analysis of nulliparous young women’s perspectives with respect to this extraordinarily complex decision. Secondly, we recruited people on social media sites that may have drawn youth that were focused on climate change more than the average youth in BC. Those who are more passionate about climate change may have been the ones who were more likely to seek out and self-select to our study. Thirdly, our study did not include the perspectives of men, non-binary, trans or Two-Spirit people with respect to childbearing and climate change. While other studies on this topic have not restricted participation based on sex or gender, our study included young adults who identified as female as birth (cis women, trans men, and non-binary people were encouraged to participate) and considered climate change in their decision to bear children. We recognize that partners of people with uteruses and their desires and needs also factor into family planning; in the next phase of our research, we plan to explore perspectives of other sexes and genders, including those assigned male at birth. Lastly, we asked participants to avoid taking photos of identifiable people. This led participants to share photos which primarily featured nature and landscapes. However, their disclosure around relationships with romantic partners, family and friends, and the role of influencers and politicians emerged naturally during interviews.

## Conclusion

With this study, we aimed to explore how climate change may impact youth decision making to have children using a novel arts-based approach to elicit emotional disclosure. Responses ranged from feeling that climate change had no effect on family planning to choosing not to have children entirely because of climate change. Most participants expressed apprehension about the future both for themselves and their potential offspring due to the environment. This study is important for future research into the complex decision of childbearing among youth and may help to direct climate change policy, specifically once the national impact of climate change on reproductive decision making is better understood. Our findings also indicated that healthcare professionals and decision-making tools involved in family planning and/or contraceptive use among young people should acknowledgement the possible influence of climate change in one’s decision whether or not to become pregnant.

## Electronic supplementary material

Below is the link to the electronic supplementary material.

Study Supplementary File I- Additional Photographs.

Supplementary File 2- Guided Interview Questions

## Data Availability

All anonymized data generated or analysed during this study are included in this published article and its supplementary information files.
