# Supplementary material for: Pregnancy intentions of young women in Canada in the era of climate change: a qualitative auto-photography study
Source: BMC Public Health. 2023 Apr 25;23:766. doi: 10.1186/s12889-023-15674-z (PMC10127979; doi:10.1186/s12889-023-15674-z)
Supplement: Supplementary file 1 — Study Supplementary File I- Additional Photographs. [file 12889_2023_15674_MOESM1_ESM.docx]

**Pregnancy Intentions of Youth in the Era of Climate Change: A Qualitative Auto-Photography Study
Supplementary File I- Additional Photographs**


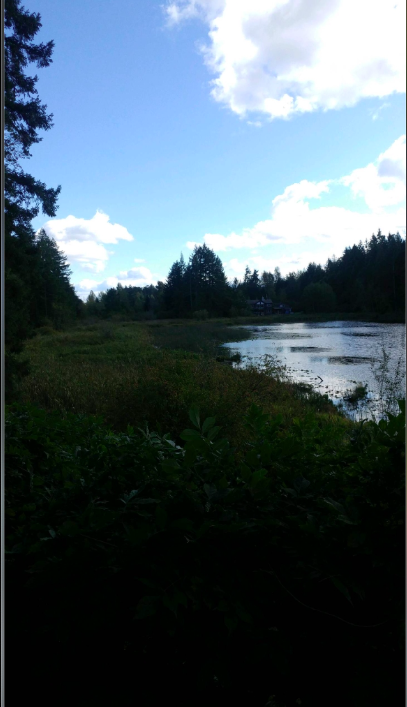


Supplementary figure 1, *Clear skies in Nanaimo,* Participant 1


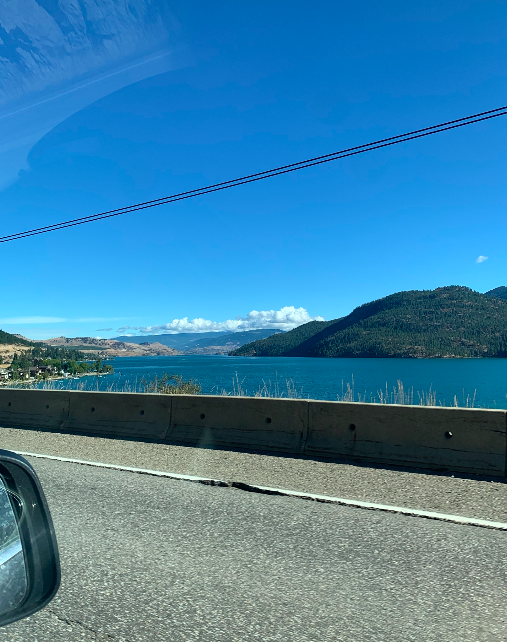


Supplementary figure 2, *Fresh water lake in the Okanagan,* Participant 1


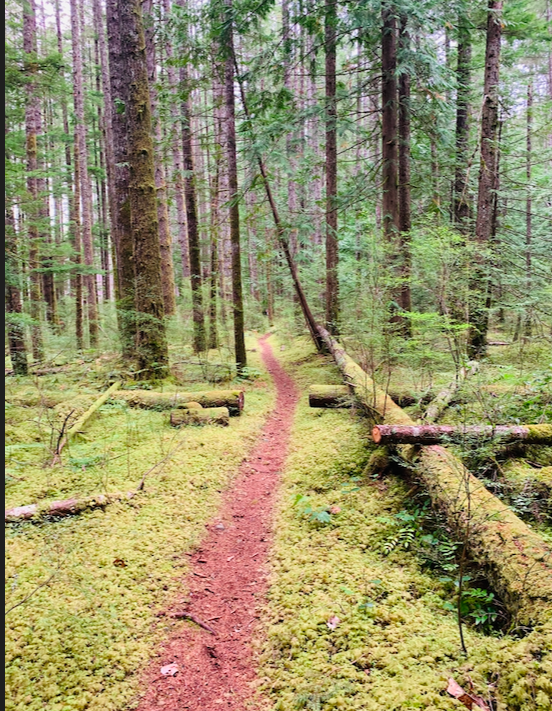


Supplementary figure 3, *Lush and green on Quadra Island*, Participant 1


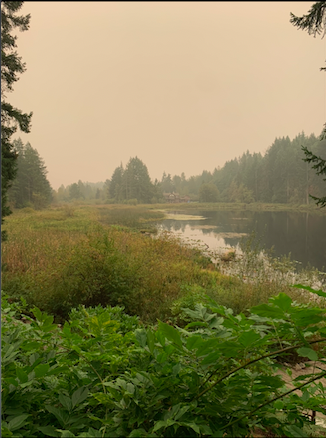


Supplementary figure 4, *Smoke in Nanaimo,* Participant 1


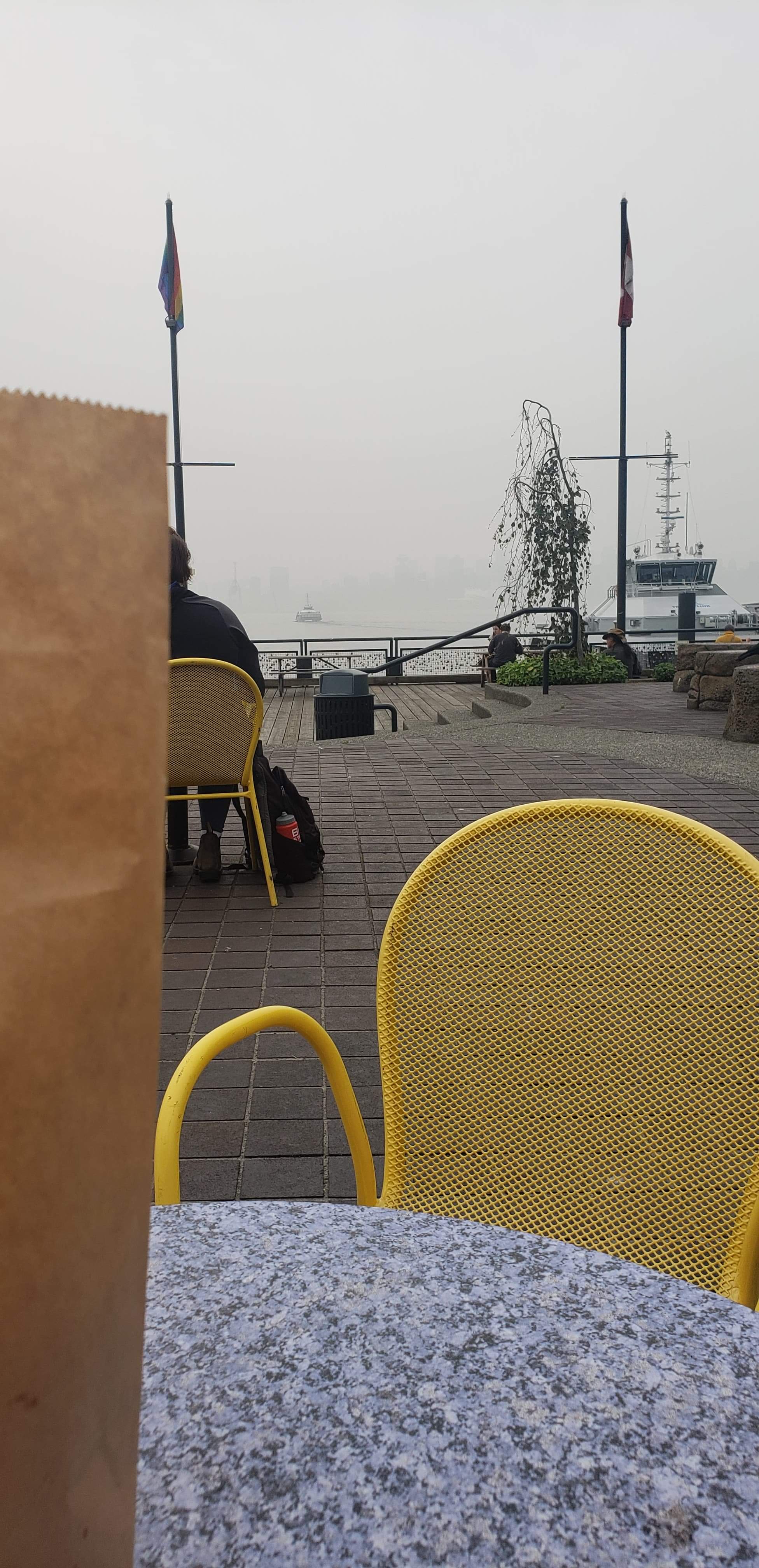


Supplementary figure 5, *Wildfire Smoke,* Participant 2


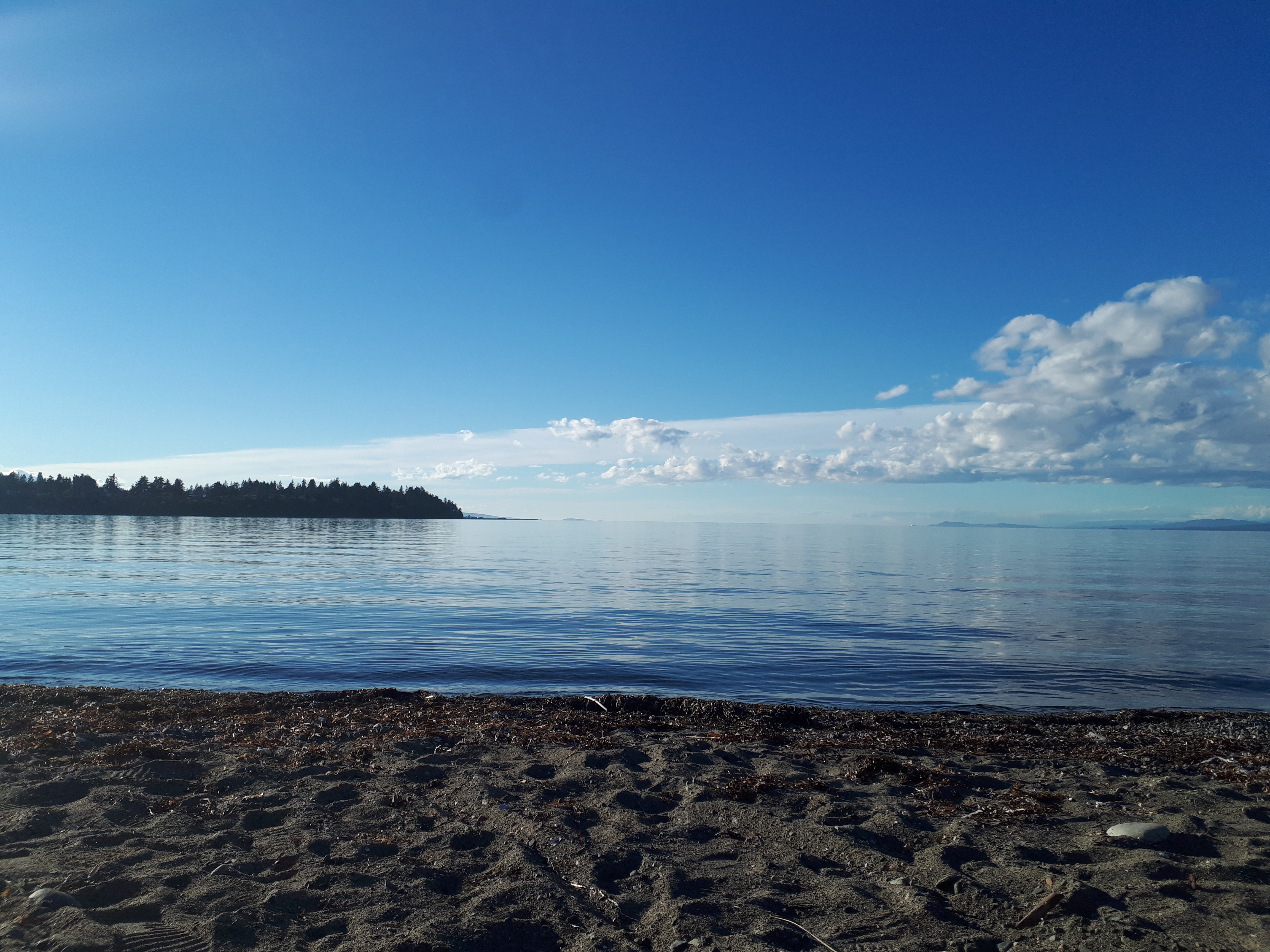


Supplementary figure 6, *High* *Tide,* Participant 2


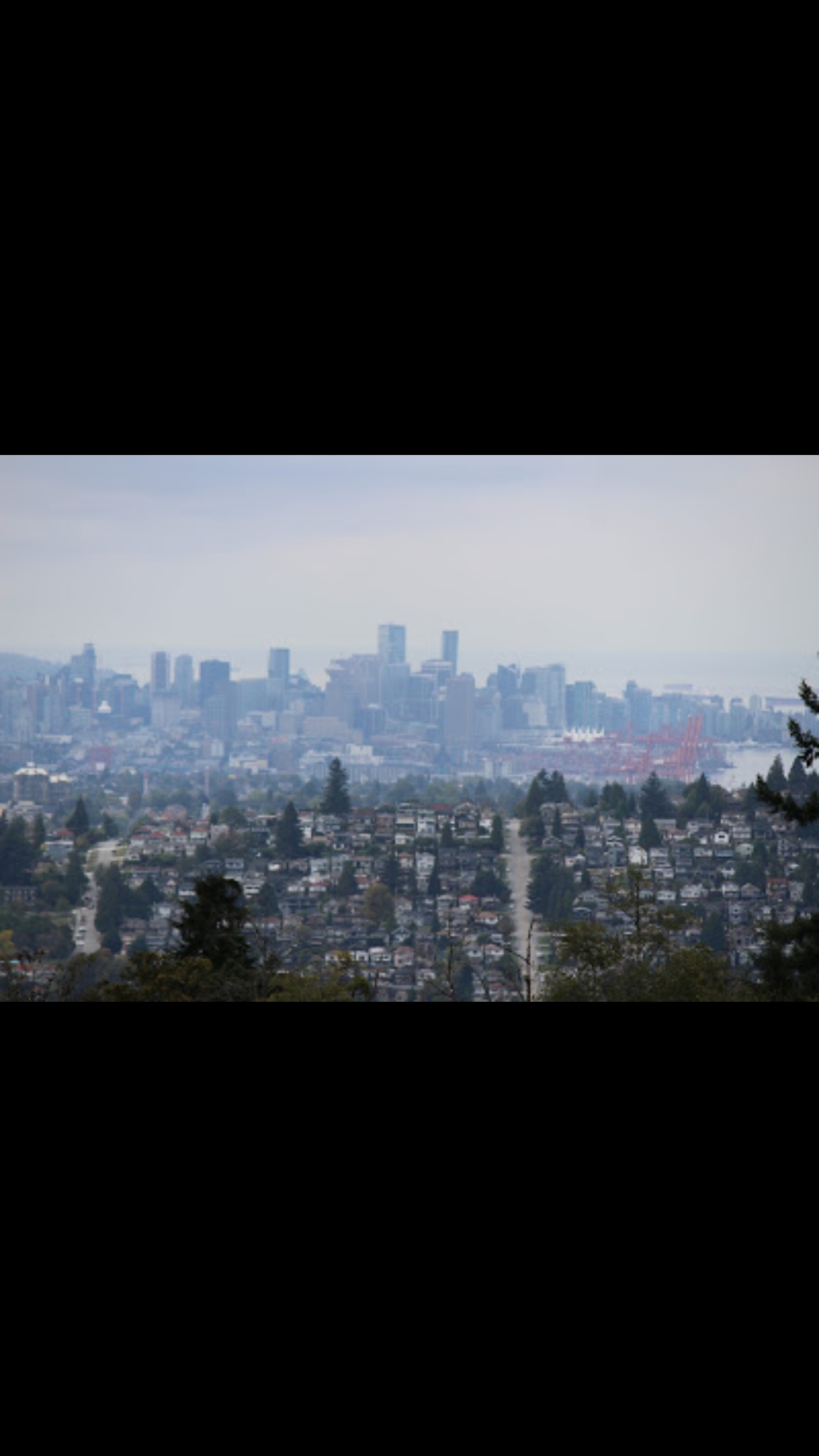


Supplementary figure 7, *Industrialization,* Participant 2


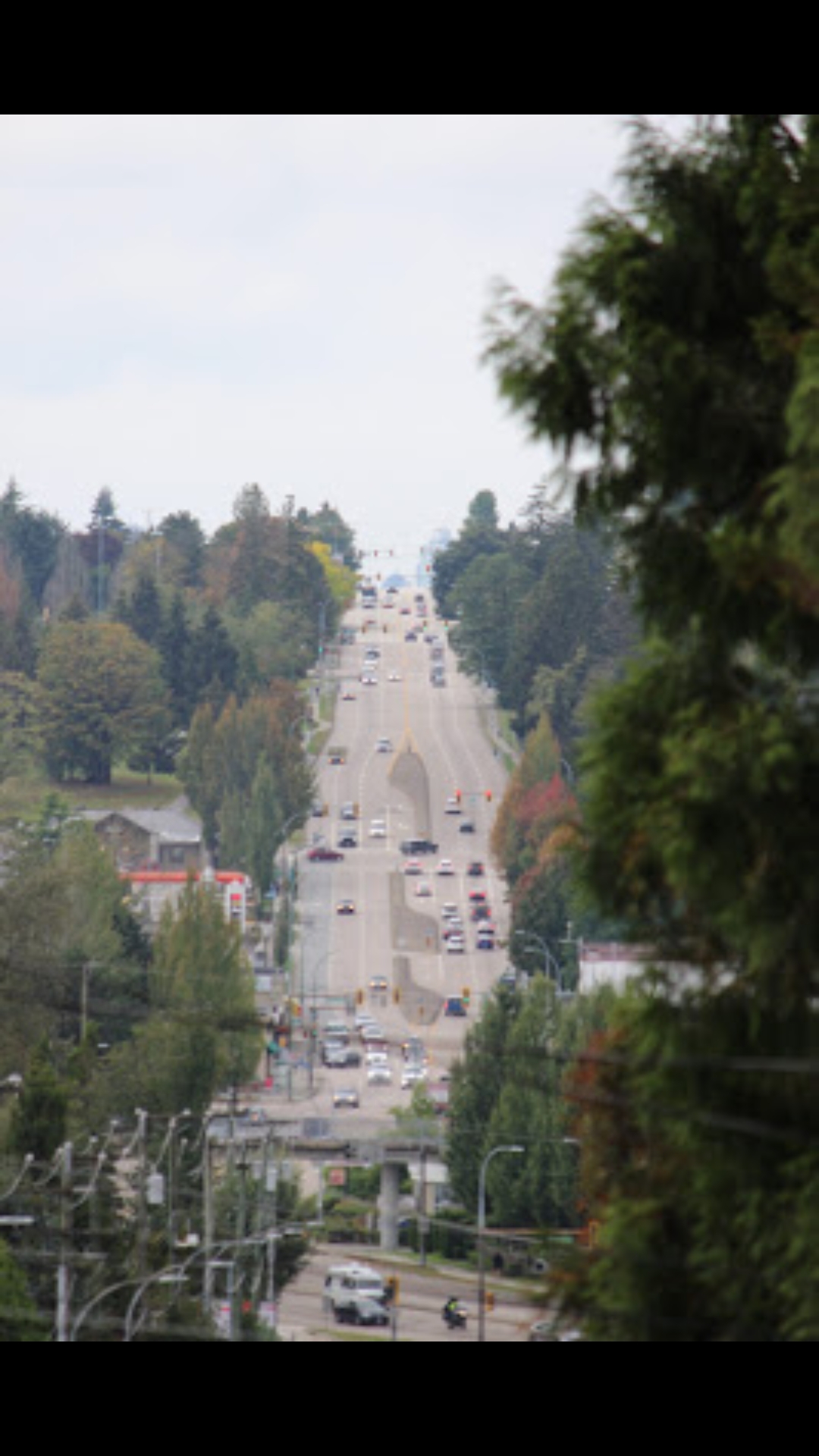


Supplementary figure 8, *Busy* *Streets,* Participant 2


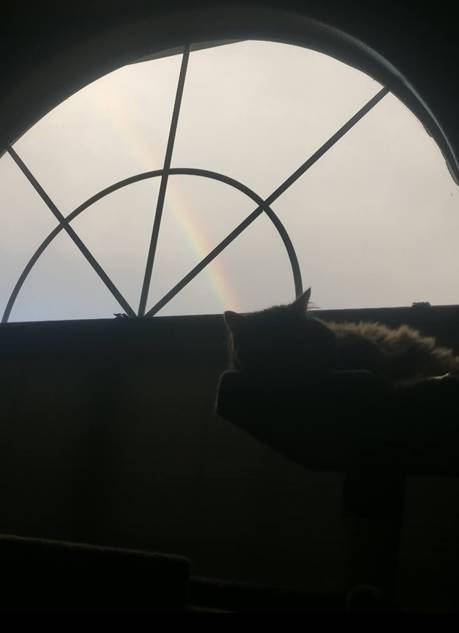


Supplementary figure 9, *Foggy Future,* Participant 3


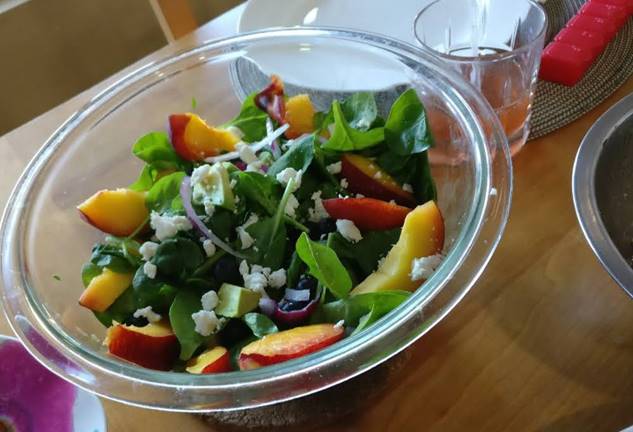


Supplementary figure 10, *We are able to teach environmentally conscious dietary choices, such as reduce consumption of meat,* Participant 3


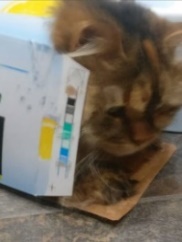


Supplementary figure 11, *Cat promoting recycling,* Participant 3

**
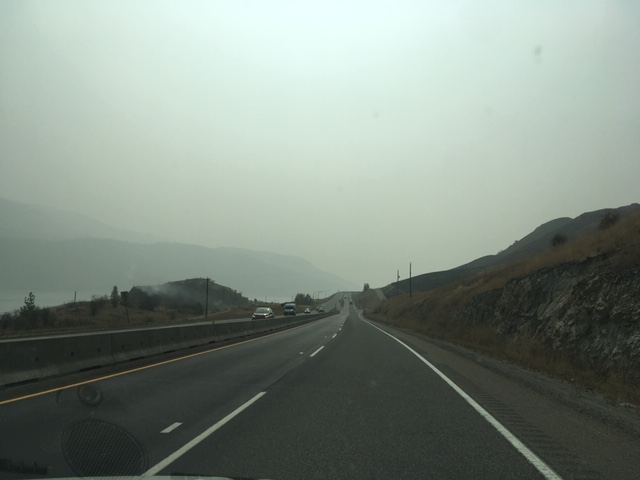
** Supplementary figure 12, *What’s up ahead?* Participant 4


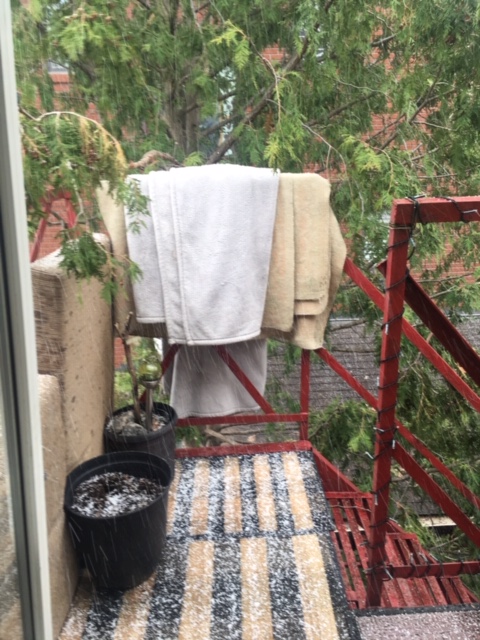


Supplementary figure 13, *There is no God,* Participant 4


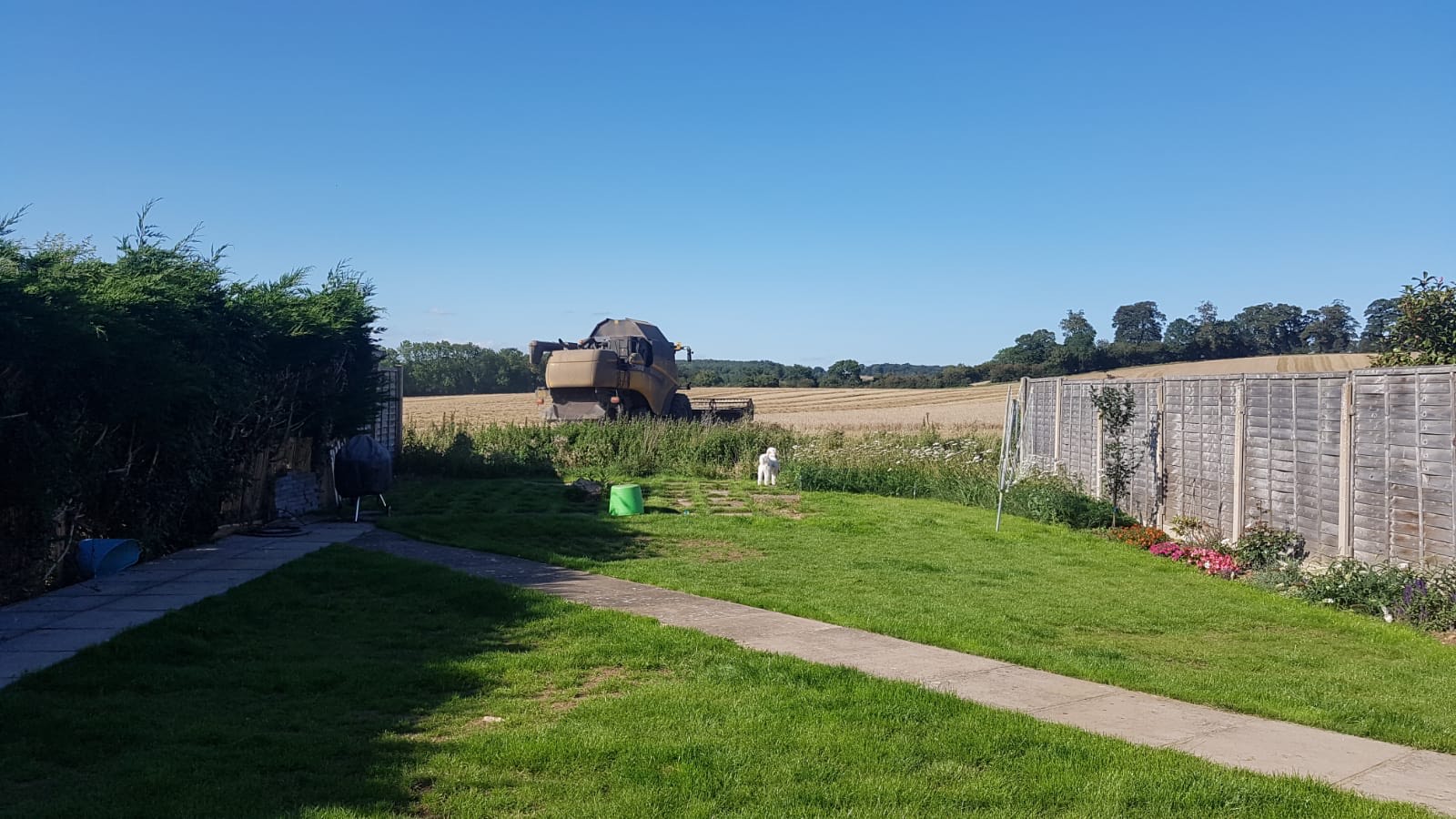
 Supplementary figure 14, *Ignorance is Bliss,* Participant 5


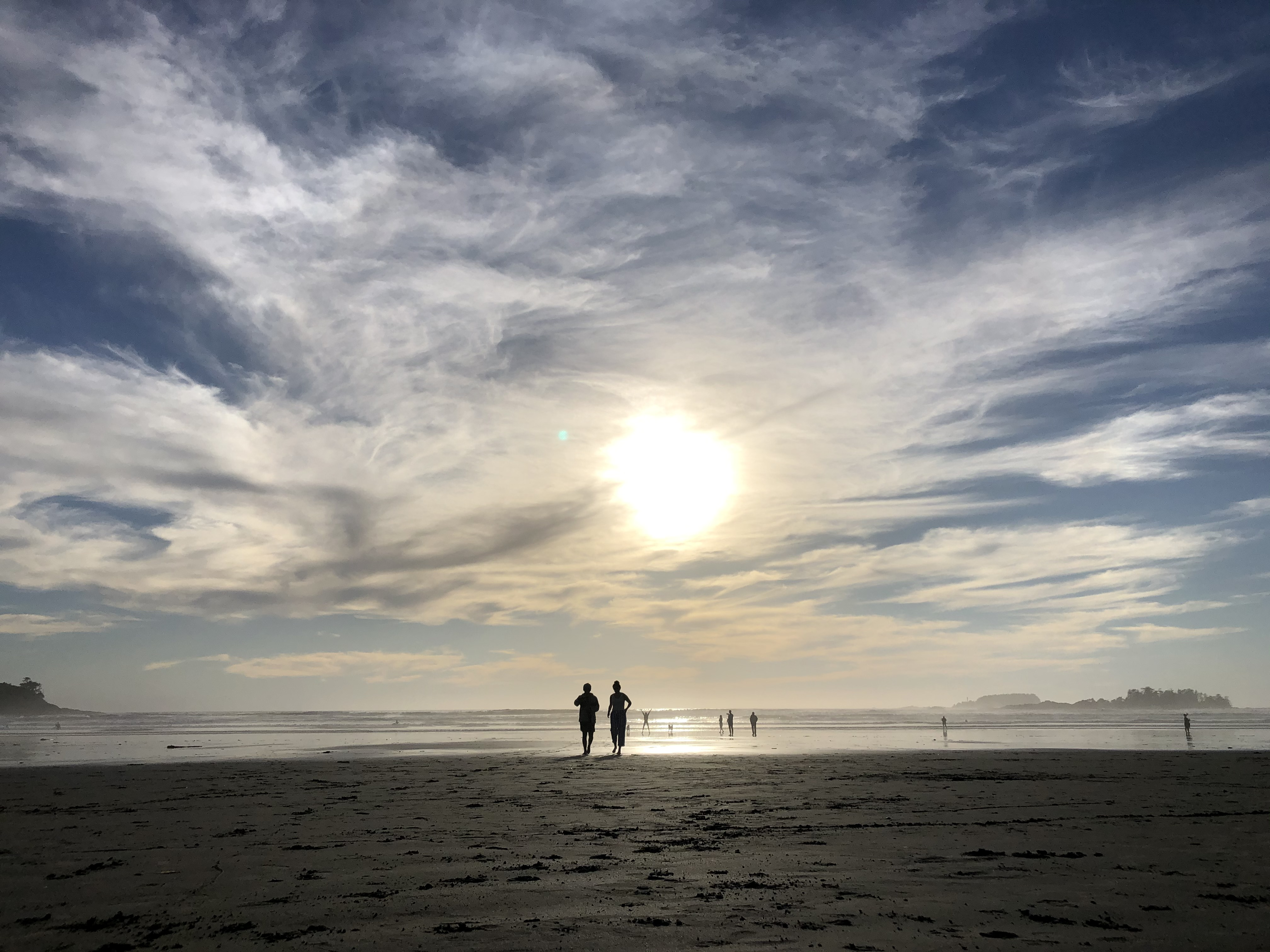


Supplementary figure 15, *Taking in the Natural Beauty,* Participant 5


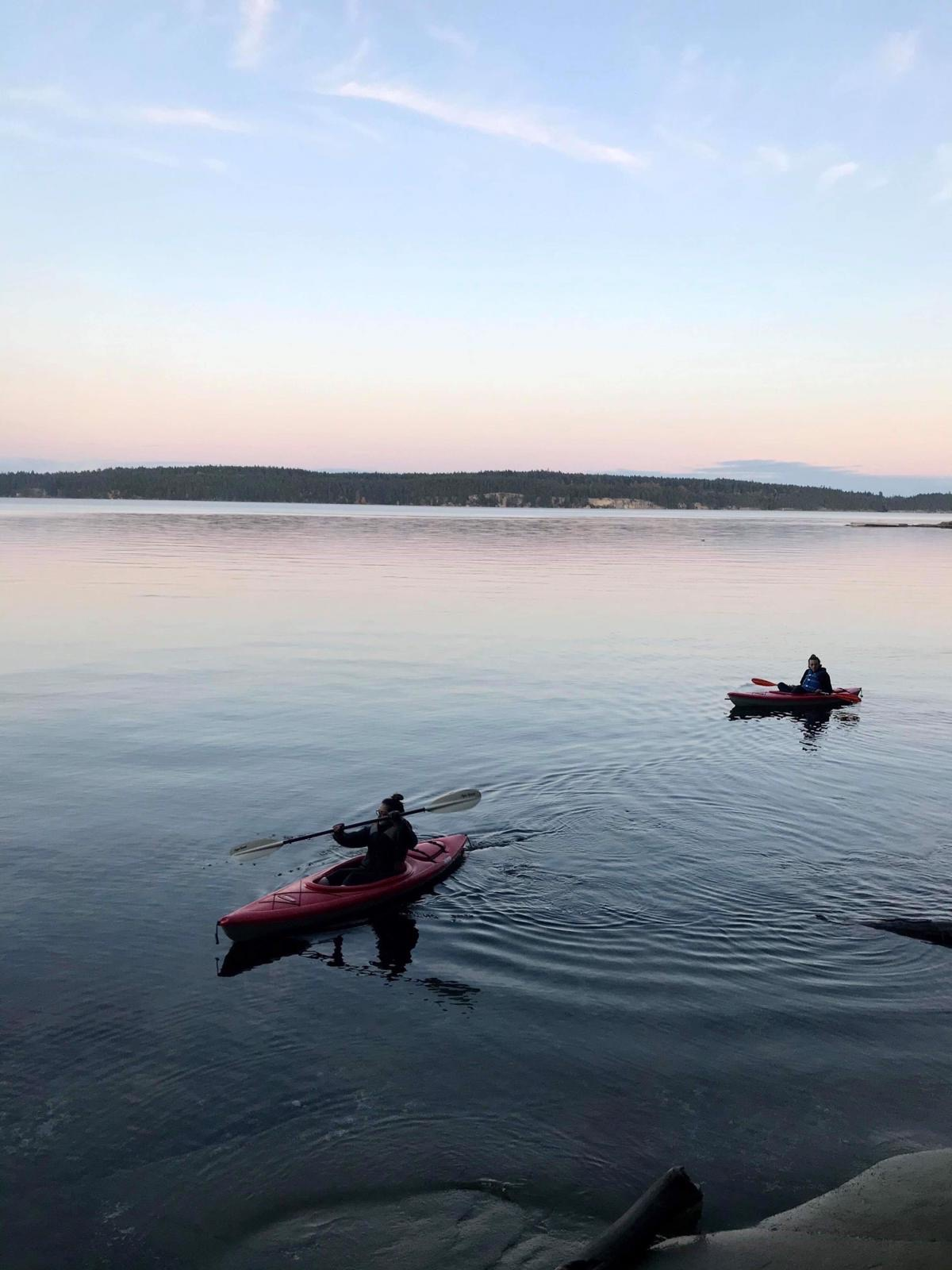


Supplementary figure 16, *Nature’s Serenity,* Participant 5


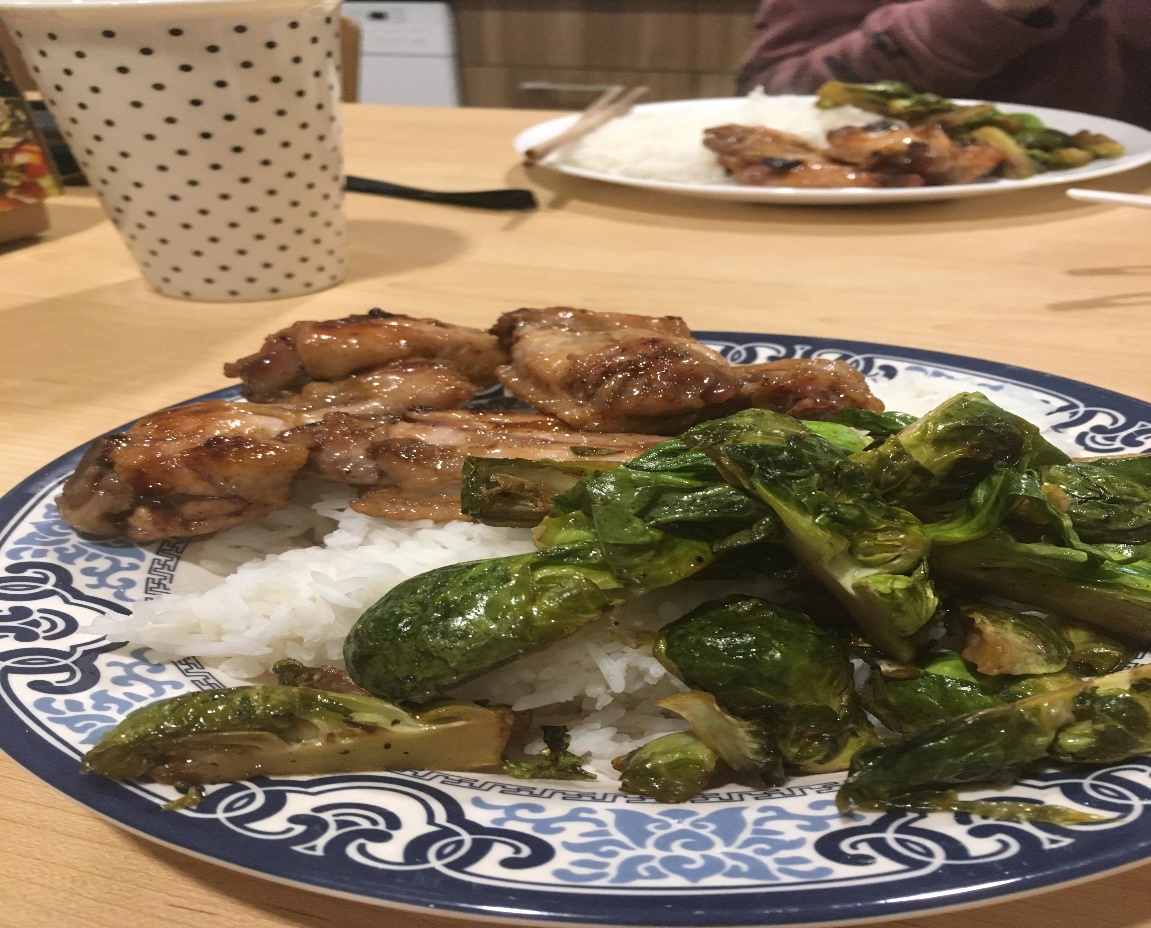


Supplementary figure 17, *Eating with Family,* Participant 6


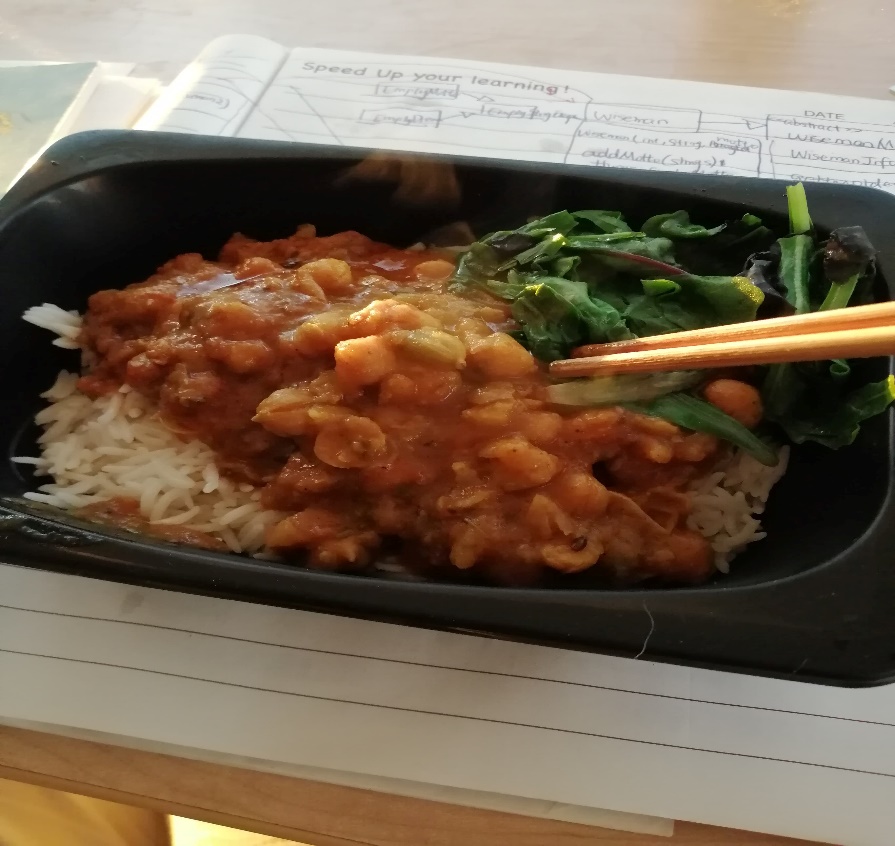


Supplementary figure 18, *Eating alone fast food (not environmental friendly),* Participant 6


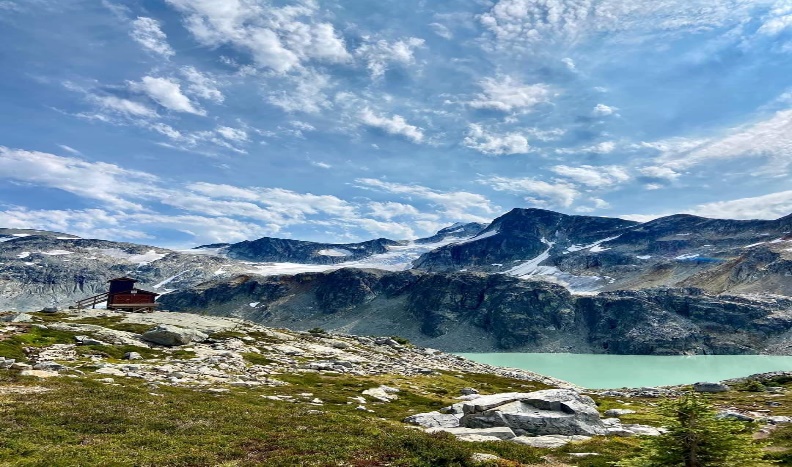


Supplementary figure 19, *Climb with my family (appreciation to the nature),* Participant 6


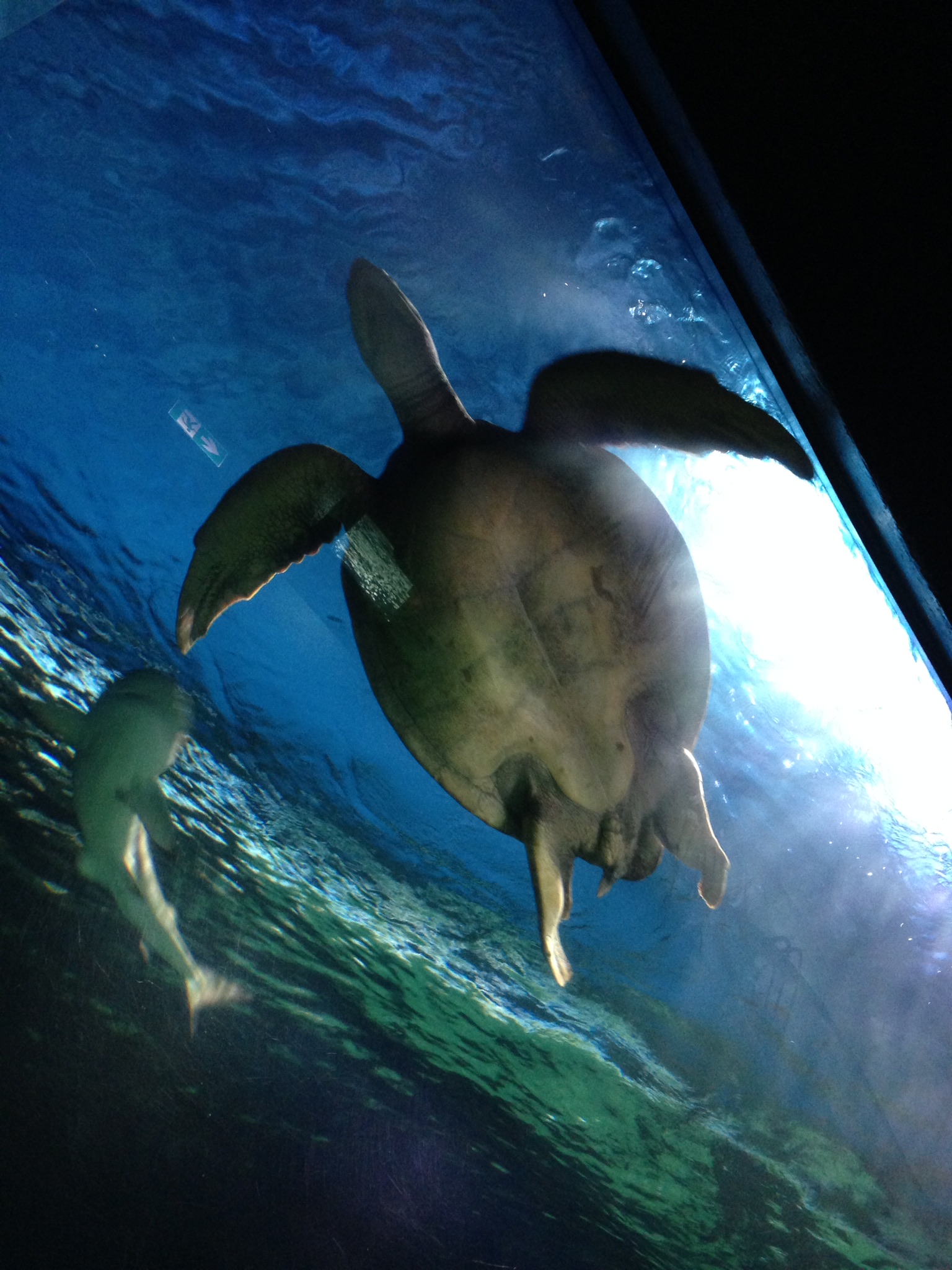


Supplementary figure 20, *Enclosed* *Beauty*, Participant 7


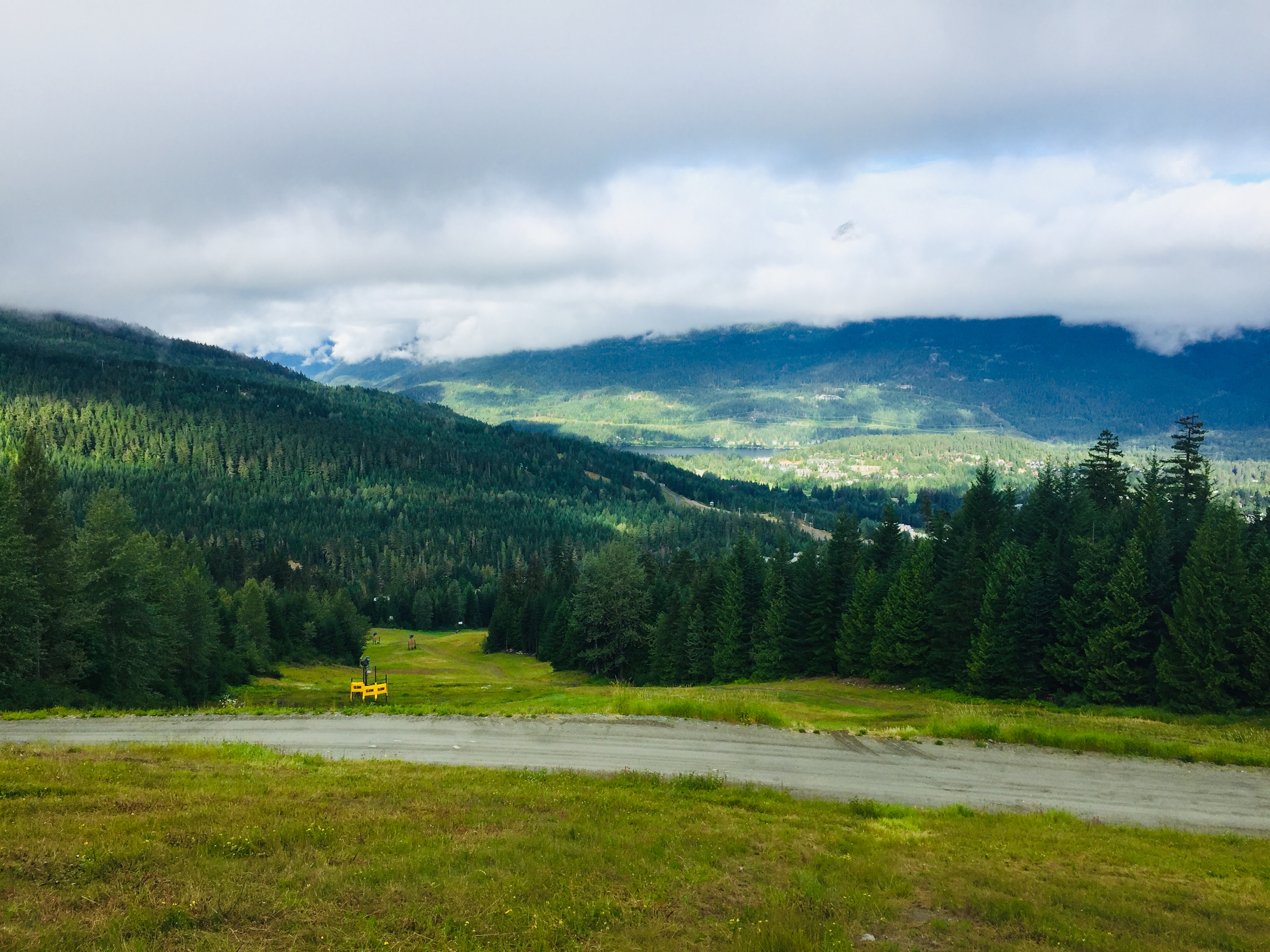


Supplementary figure 21, *No Title,* Participant 7


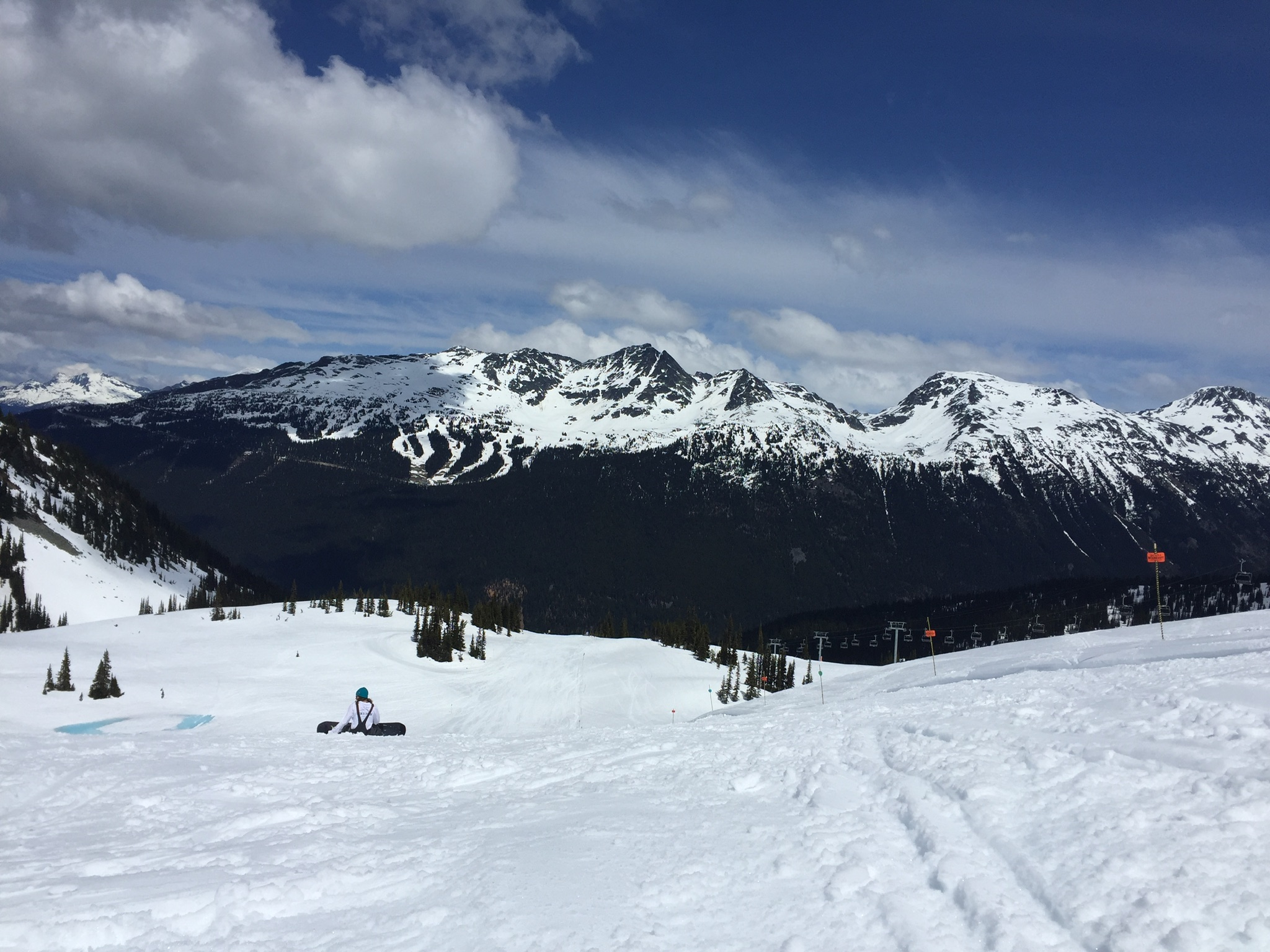


Supplementary figure 22, *No* *Title,* Participant 7
