# Supplementary material for: Pregnancy intentions of young women in Canada in the era of climate change: a qualitative auto-photography study
Source: BMC Public Health. 2023 Apr 25;23:766. doi: 10.1186/s12889-023-15674-z (PMC10127979; doi:10.1186/s12889-023-15674-z)
Supplement: Supplementary file 2 — Guided Interview Questions [file 12889_2023_15674_MOESM2_ESM.docx]

**Pregnancy Intentions of Youth in the Era of Climate Change: A Qualitative Auto-Photography Study
Supplementary File 2- Guided Interview Questions**

1. Please describe what is in each photograph.
2. Why did you take this particular picture?
3. Talk about the meaning of the objects in the photo.
4. How do you feel when you look at what is pictured?
5. If you could create a title for this picture, what would it be?
6. What are your plans for having children?

When did you first feel this way?

Can you give me an example about when you first questioned your decision to have a family?

In summary, can you explain how this photo impacts your preferences to have children

1. If you could change one thing about this photo that would alter your decision to have a family, what would it be?
